# Celastrol Combats Methicillin‐Resistant *Staphylococcus aureus* by Targeting Δ^1^‐Pyrroline‐5‐Carboxylate Dehydrogenase

**DOI:** 10.1002/advs.202302459

**Published:** 2023-06-28

**Authors:** Zhongwei Yuan, Jun Wang, Qianwei Qu, Zhenxin Zhu, Marc Xu, Mengmeng Zhao, Chongxiang Sun, Haixin Peng, Xingyu Huang, Yue Dong, Chunliu Dong, Yadan Zheng, Shuguang Yuan, Yanhua Li

**Affiliations:** ^1^ Heilongjiang Key Laboratory for Animal Disease Control and Pharmaceutical Development College of Veterinary Medicine Northeast Agricultural University Harbin 150030 China; ^2^ Shenzhen Institutes of Advanced Technology Chinese Academy of Sciences Shenzhen 518055 China

**Keywords:** celastrol, MRSA, multi‐omics, multiple pathways, P5CDH

## Abstract

The emergence and rapid spread of methicillin‐resistant *Staphylococcus aureus* (MRSA) raise a critical need for alternative therapeutic options. New antibacterial drugs and targets are required to combat MRSA‐associated infections. Based on this study, celastrol, a natural product from the roots of *Tripterygium wilfordii* Hook. f., effectively combats MRSA in vitro and in vivo. Multi‐omics analysis suggests that the molecular mechanism of action of celastrol may be related to Δ^1^‐pyrroline‐5‐carboxylate dehydrogenase (P5CDH). By comparing the properties of wild‐type and *rocA‐*deficient MRSA strains, it is demonstrated that P5CDH, the second enzyme of the proline catabolism pathway, is a tentative new target for antibacterial agents. Using molecular docking, bio‐layer interferometry, and enzyme activity assays, it is confirmed that celastrol can affect the function of P5CDH. Furthermore, it is found through site‐directed protein mutagenesis that the Lys205 and Glu208 residues are key for celastrol binding to P5CDH. Finally, mechanistic studies show that celastrol induces oxidative stress and inhibits DNA synthesis by binding to P5CDH. The findings of this study indicate that celastrol is a promising lead compound and validate P5CDH as a potential target for the development of novel drugs against MRSA.

## Graphical abstract

Schematic diagram summarizing the antibacterial mechanisms of celastrol against MRSA. Celastrol activates multiple pathways including inhibiting DNA and protein biosynthesis, generating ROS and inducing cell death by competitively binding to P5CDH.

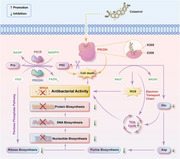

## 1 Introduction

*Staphylococcus aureus* (*S. aureus*) is an opportunistic, Gram‐positive bacterium that causes several different infections in humans and animals, ranging from skin and soft tissue infections to sepsis and meningitis.^[^ ^1^, ^2^ ^]^ The widespread, indiscriminate use of antibiotics for bacterial infections worsens drug resistance. The emergence and spread of multidrug‐resistant *S. aureus* isolates such as methicillin‐resistant *S. aureus* (MRSA) poses a threat to public health worldwide. Notably, the deaths caused by MRSA exceed those caused by acquired immune deficiency syndrome, tuberculosis, and viral hepatitis.^[^ ^3^ ^]^ Unfortunately, the development of a traditional antibacterial agent requires, on average, an investment of 1.5 billion dollars and more than 10 years, leading many enterprises to abandon the development of antibacterial drugs.^[^ ^4^ ^]^ Therefore, there is an urgent need to discover novel targets and drugs to combat MRSA infections.

**Figure 1:**
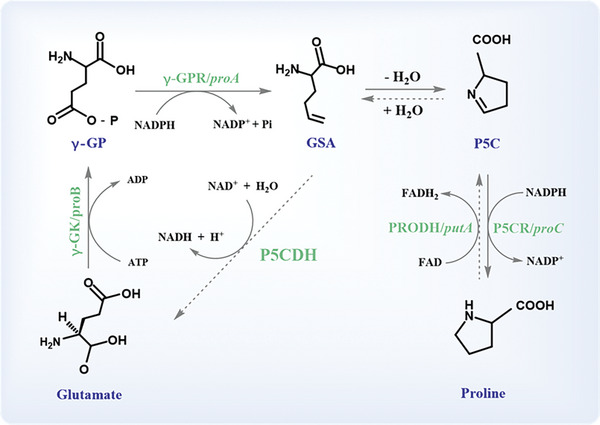
Biosynthesis and degradation of Pro in bacteria.

Δ^1^‐pyrroline‐5‐carboxylate dehydrogenase (P5CDH, encoded by the *rocA* gene) is an enzyme in the second part of the conversion of proline (Pro) to glutamate (Glu), which catalyzes Δ^1^‐pyrroline‐5‐carboxylate (P5C) into Glu^[^ ^5^ ^]^ (**Figure** **1**). As an intermediate of Pro, Glu, and ornithine interconversions, P5C links the TCA cycle, urea cycle, and Pro metabolism.^[^ ^6^, ^7^, ^8^ ^]^ Meanwhile, P5C is coupled to the pentose phosphate pathway, influencing nucleotide production for DNA biosynthesis via NADP^+^/NADPH.^[^ ^9^ ^]^ Notably, P5C can be degraded in two pathways: I) converting to Glu by P5CDH (main mode); II) conversion to Pro by P5C reductase. Thus, the intracellular P5C content is affected by P5CDH. Taken together, P5CDH participates not only in the regulation of survival processes—growth and proliferation, via involvement in the synthesis of biomass components—but also in the regulation of cell death, due to the modulation of reactive oxygen species (ROS) levels. Importantly, a mutant of P5CDH could influence the growth of bacteria.^[^ ^10^, ^11^ ^]^ Therefore, we speculate that P5CDH is a promising target for the development of antibacterial drugs.

Plants account for the most biomass on our planet.^[^ ^12^ ^]^ Plant‐derived compounds and natural products have been widely used to treat different diseases.^[^ ^13^ ^]^ Recently, by analyzing more than 100 natural products from plants known to exhibit antibacterial activity against MRSA, we found that celastrol represents a promising antibacterial lead compound. Celastrol is a pharmacologically active pentacyclic triterpene extract from the roots of the traditional Chinese herbal plant *Tripterygium wilfordii* Hook. f.^[^ ^14^ ^]^ As early as 2007, celastrol has been suggested as one of five candidate compounds showing potential as a therapeutic agent.^[^ ^15^ ^]^ Recently, its leptin‐sensitizing and anti‐obesity effects have attracted worldwide attention.^[^ ^16^, ^17^, ^18^ ^]^ To date, celastrol has been reported to have potential in the treatment of obesity, liver fibrosis, arthritis, and cancer.^[^ ^18^, ^19^, ^20^, ^21^ ^]^ However, there are no studies available on its antibacterial properties, especially against MRSA, till now. In this study, we found that celastrol had excellent anti‐MRSA activity and reasoned that the antibacterial target of celastrol may be P5CDH from multi‐omics (transcriptomics, proteomics, and metabolomics) analysis. In this study, we evaluated the therapeutic effects of celastrol in vitro and in vivo and systematically studied the anti‐MRSA mechanism of its targeting of P5CDH. Overall, our findings suggest that celastrol can affect the enzyme activity of P5CDH against MRSA, which provides the basis and strategies for developing new anti‐MRSA infection agents.

## 2 Experimental Section

### 2.1 Bacteria and Reagents

All strains used in this study are listed in Table S1 (Supporting Information). Celastrol (>98% purity, HPLC; CAS No. 34157‐83‐0) was purchased from Chengdu Herbpurify Co., Ltd. (Chengdu, China). A stock solution of celastrol (40.96 mg mL^−1^) was prepared in dimethyl sulfoxide (DMSO). Antibiotics namely linezolid, oxacillin, vancomycin, chloramphenicol, kanamycin, and ampicillin were purchased from Shanghai Yuanye Bio‐Technology Co., Ltd (Shanghai, China). Triton X‐100, 10% formalin, and 4% paraformaldehyde were purchased from Beijing Leagene Biotechnology Co., Ltd (Beijing, China).

### 2.2 Antimicrobial Susceptibility Testing

The broth microdilution method was used to measure the minimal inhibitory concentration (MIC) of celastrol as described by the Clinical and Laboratory Standards Institute,^[^ ^22^ ^]^ and then the bacterial viability was examined using 0.1% (w/v) resazurin (Coolaber, Beijing, China). The lowest concentration of celastrol that inhibited the growth was defined as the MIC. Minimum bactericidal concentration (MBC) was calculated by removing 100 µL from each well that did not exhibit visual bacteria growth and then subculturing on agar plates followed by incubation at 37 °C for 24 h.^[^ ^23^ ^]^

### 2.3 Growth Curve

The growth curve was generated according to the previous study.^[^ ^24^ ^]^ Briefly, the bacterial suspensions of MRSA strains were treated with different concentrations of celastrol at 37 °C with continuous shaking (160 rpm). At various time points, the OD_600 nm_ of a 1‐mL aliquot was measured using a UV‐vis spectrophotometer (UNICO, Shanghai, China).

### 2.4 Time‐Dependent Killing Curve

The bacterial suspensions of MRSA USA300 were adjusted in tryptic soy broth (TSB) to 10^6^ CFU mL^−1^. Then, the bacterial suspensions were treated with different concentrations of celastrol at 37 °C with continuous shaking (160 rpm). The samples were removed at 1, 2, 4, 8, and 24 h to count the number of surviving bacteria with TSA plates.^[^ ^25^ ^]^

### 2.5 Bacterial Resistance Studies

For resistance development by sequential passaging, MRSA USA300 at the exponential phase was passaged to new MHB containing celastrol, oxacillin, or vancomycin at concentrations of 0.5× MIC. After being incubated in a shaker (160 rpm, 37 °C) for 24 h, the bacterial suspensions were then re‐passaged for the next MIC assay. The process was repeated for 30 passages.^[^ ^25^ ^]^

### 2.6 *G. mellonella* Larvae Infection Model

Larvae of (*Galleria mellonella*) *G. mellonella* (Huiyude Biotech Company, Tianjin, China) were randomly divided into six groups (*n* = 10 per group), of which five groups were infected with MRSA USA300 suspension (1.0 × 10^7^ CFU mL^−1^) at the right posterior gastropoda. After 2 h of infection, *G. mellonella* larvae were administered PBS, celastrol (5, 10, or 20 mg kg^−1^), and vancomycin hydrochloride (20 mg kg^−1^) at the left posterior gastropoda. The remaining group was injected with PBS only as a blank group. The volume of all injections was 10 µL. Survival rates of *G. mellonella* larvae were recorded over the following 72 h.^[^ ^26^ ^]^

### 2.7 Animal Studies

Female 8‐week‐old BALB/c mice (weighing 18–20 g) from the Experimental Animal Center of the Second Affiliated Hospital of Harbin Medical University (Harbin, China) were used in this study. Mice were adapted to standardized environmental conditions for 1 week before infection at the Northeast Agricultural University to minimize potential confounders. All animal experiments were approved by the Experimental Animal Research Ethics Committee of Northeast Agricultural University (SRM‐11).

#### 2.7.1 Skin MRSA Infection

The mouse model of skin infection was established according to the method described by Malachowa et al.^[^ ^27^ ^]^ One day after infection, the mice were randomly divided into five groups (*n* = 5 per group). In the antibiotic‐treated group, the mice were treated with vancomycin hydrochloride (0.4 mg kg^−1^). Mice in the three celastrol treatment groups were administered 0.1, 0.2, and 0.4 celastrol mg kg^−1^, respectively. In the control group, the mice were administered isotonic sterile PBS. All drugs and PBS were administered to the wounds twice a day. The volume of all injected drugs was 0.05 mL. At the end of the experiment, the size of the wound skin was measured, and a portion of the wound skin was excised for homogenization to determine the bacterial burdens. The remaining portion of the wound skin was sectioned with a microtome and stained with hematoxylin and eosin (H&E) for microscopic observation.

#### 2.7.2 MRSA‐Induced Bacteremia

A mouse model of MRSA‐induced bacteremia was established according to the method described by Wang.^[^ ^28^ ^]^ Briefly, BALB/c mice were inoculated with 100 µL of staphylococcal suspension (2 × 10^8^ CFU mL^−1^) into the tail vein. After 30 min, the mice were randomly divided into five groups (*n* = 5 per group) and were treated with celastrol (6.25 mg kg^−1^), celastrol (12.5 mg kg^−1^), celastrol (25 mg kg^−1^), linezolid (25 mg kg), and PBS via oral treatment twice a day. On day 6 after infection, the mice were euthanized and their kidneys and livers were excised. The kidneys and livers were homogenized and diluted in PBS, and the bacterial burdens in the kidneys were calculated by colony counting. The hearts, livers, spleens, lungs, and kidneys from each group of mice were fixed in 10% formalin. After processing, the tissues were sectioned with a microtome and stained with hematoxylin and eosin (H&E) for microscopic observation.

### 2.8 Multi‐Omics (Transcriptomics, Proteomics, and Metabolomics) Analysis

#### 2.8.1 Sample Preparation

MRSA USA300 cells were cultured to logarithmic phase and treated with 1/2 MIC celastrol (0.5 µg mL^−1^) or an equal volume of DMSO for 4 h in TSB. The bacterial culture was collected by centrifugation (5500 rpm, 5 min) and washed with PBS twice. Samples were obtained in triplicate for transcriptomic, proteomic, and metabolomic analyses. Six samples were obtained for each concentration. The subsequent operation and processing of the three omics were performed by Shanghai iProteome Biotechnology Co., Ltd (Shanghai, China).

#### 2.8.2 RNA Sequencing

The TruSeqTM Stranded Total RNA Library Prep Kit (Illumina, USA) was used to construct (RNA‐seq) libraries for RNA sequencing. RNA quality and concentration were initially analyzed using NanoDrop spectrophotometers 2000 (Thermo Fisher Scientific, USA). Furthermore, RNA integrity was assessed using agarose gel electrophoresis and Agilent Bioanalyzer 2100 (Agilent Technologies, USA). After Illumina sequencing, the data were analyzed, and statistical significance was defined at *p* < 0.05.

#### 2.8.3 Label‐Free LC–MS/MS Proteomics Analysis

Protein was extracted from samples by 8 m urea and quantified by NanoDrop spectrophotometers 2000. For each sample, 60 µg of protein was trypsinized using filter‐aided sample preparation. The peptides were lyophilized and stored at −80 °C. After separation by HPLC liquid system EASY‐nLC1200 (Thermo Fisher Scientific, USA), the samples were analyzed by mass spectrometry using an OE480 mass spectrometer (Thermo Fisher Scientific, USA). The raw data were retrieved, identified, and analyzed quantitatively using the Firmiana proteomic cloud platform.^[^ ^29^ ^]^ The differentially expressed proteins were filtered by the following cutoff criteria: the *p*‐value for the *t*‐test was <0.05 and the screening differential multiple was >2. BLAST2GO and KOBAS were used to annotate proteins with GO and KEGG, respectively.

#### 2.8.4 Metabolomic Analysis

For untargeted metabolomics analysis, cells were added with methanol and ultrasonicated in an ice bath for 10 min. Then samples were maintained at −20 °C for 1 h to precipitate protein followed by centrifugation (14 000 rpm, 10 min, and 4 °C). The supernatant was dried in a vacuum at 20 °C and stored at −80 °C. Samples were prepared for metabolon‐based energy metabolism detection using a Nexera UHPLC LC‐30A (SHIMADZU, Japan) coupled to a QTRAP (Thermo Fisher Scientific, USA). For detection, first, metabolite features were detected and screened in both negative and positive ion modes. Subsequently, the internal standard normalization method was employed for the data analysis. The resulting 3D data including the peak number, sample name, and normalized peak area were fed into the R package metaX for principal component analysis and orthogonal partial least square‐discriminate analysis.

### 2.9 Constructions of rocA Knockout Bacteria

The *rocA* knockout strain of MRSA USA300 was constructed using plasmid pKOR1. Briefly, the upstream and downstream fragments of *rocA* were amplified from the genomic DNA of MRSA USA300. The PCR products were cloned into the pKOR1 by T4 DNA Ligase (Takara); the constructed plasmid was named pKOR1‐*rocA*. After being modified by RN4220, the plasmid was transduced into the MRSA USA300 using phage transduction. The knockout cells were detected on a TSA plate containing 10 µg mL^−1^ chloramphenicol. Finally, the genome editing was confirmed by PCR using the extracted genomic DNA and further verified by DNA sequencing. In addition, the plasmid in the mutant strain was cured according to a previously described protocol.^[^ ^30^ ^]^

### 2.10 Construction of rocA Complemented Strain

To complement the mutation of the *rocA* mutant, the complete *rocA* gene from MRSA USA300 was PCR amplified and cloned into a single copy plasmid vector pCM28 (GenBank: MN956986.1). After being transformed by electroporation into the RN4220 strain, the recombinant plasmid was transduced into the mutant strains by the same method mentioned above. The complemented gene in the mutant was verified by performing PCR and DNA sequencing.

### 2.11 Homology Modeling of P5CDH Protein and Molecular Docking of Compounds

Since the 3D structure of the P5CDH protein from MRSA was not available in the PDB database (https://www.rcsb.org/), the homology modeling technique was used for docking and structure‐based design. After the final model evaluation, molecular docking was performed using the Schrodinger Glide (Schrödinger Release 2021–4: Glide, Schrödinger, LLC, New York, NY, 2021.). The putative protein structure was preprocessed by the Protein Preparation Wizard module from Schrodinger to remove potential atom clashes. Both protein‐ligand structures were refined with an OPLS4 force field and protonated at pH 7. The ligand diameter midpoint was enclosed to 40 Å in three‐axis and the box size was set to 36 Å to include entirely all putative binding spaces. The box center was placed at the center of the protein (6.33, 22.64, and 14.6 on the *x*, *y*, and *z* axis respectively). None of the constraints were applied. The scaling factor of 0.8 and the partial charge of 0.15 were applied according to van der Waal's law. The precision was set as default. The ligand was sampled in a flexible state, while the receptor remained rigid state.

### 2.12 Expression and Purification of P5CDH

The P5CDH protein was obtained using pET30a. The *rocA* gene, encoding the P5CDH protein, was synthesized by Integrated DNA Technologies based on its sequence in the MRSA USA300. The PCR product was ligated to pET30a via In‐Fusion cloning. The recombinant plasmid was transformed into DH5*α* competent cells. Next, plasmids were collected from DH5*α* and were transformed into BL21‐competent cells. Isopropyl‐beta‐d‐thiogalactopyranoside was added to a final concentration of 1 mm for inducing the expression of P5CDH protein in BL21. The expressed protein was purified using a Ni‐NTA agarose column and an AKTA Pure Protein Purification System (GE, USA) from the harvested bacterial suspension. Then, the quality and quantity of purified recombinant P5CDH were analyzed on 10% SDS‐PAGE gel electrophoresis.

### 2.13 Expression and Purification of P5CDH's Site‐Directed Mutant Protein

The pET30a‐P5CDH mutant was made by introducing a point mutation (Lys205, Glu208, and Asp209 to Ala) via overlap PCR and subsequently verified by sequencing (BGI). The vectors were subsequently cloned into a pET30a vector for protein expression. For expressed proteins, insoluble material was removed by centrifugation and cell‐free extracts were purified using a Ni‐NTA agarose column and an AKTA Pure Protein Purification System. Thereafter, the quality and quantity of purified proteins were analyzed using 10% SDS‐PAGE.

### 2.14 Interactions of Celastrol with P5CDH and Its Site‐Directed Mutant Protein by Bio‐Layer Interferometry (BLI)

The BLI was performed as previously described by Qu.^[^ ^31^ ^]^ Briefly, the BLI experiments were performed using an Octet system (Pall, USA) placed in PBS pH 7.4, 0.05% (v/v) Tween‐20 (Coolaber, Beijing, China), and 1 mg mL^−1^ BSA running buffer at 25 °C. Freshly prepared P5CDH protein (50 µg mL^−1^) was coupled to the tip of a Forte Bio Octet NTA instrument. A dilution series of celastrol (15 625 to 250 000 nm) was used to measure the dose‐response curve of association and dissociation. The dissociation period was set at 60 s.

### 2.15 Cellular Thermal Shift Assay

Cellular thermal shift assay (CETSA) was employed to examine the interaction between celastrol and P5CDH, as previously described.^[^ ^32^ ^]^ Briefly, the samples were equally split into two tubes, one treated with celastrol (2 µg mL^−1^) and the other with DMSO, at 25 °C for 10 min. Then the mixed solution was separated into PCR tubes, set at specific temperatures (25, 37, 42, 47, 52, 57, 62, 67, and 72 °C), and placed in a Mastercycler nexus gradient (Eppendorf) for heating. After heating for 4 min, the tubes were immediately cooled on ice for 3 min. The samples were then analyzed by western blot.

### 2.16 CD Spectroscopy

CD spectroscopy measurements were performed on a Chirascan (Applied Photophysics, UK) using a quartz cylindrical cell (path length 0.1 cm, Hellma Analytics), with P5CDH (0.1 mg mL^−1^) and celastrol (32 µg mL^−1^). Scans were conducted at 25 °C at wavelengths ranging from 185 to 260 nm. Spectra were corrected using the CD of 32 µg mL^−1^ celastrol as a baseline.

### 2.17 IC_50_ Assays

The IC_50_ values of P5CDH and its mutant proteins were measured using the delta‐1‐pyrroline‐5‐carboxylate dehydrogenase (P5CDH) kit (FT‐M52211, FanTai Biotechnology, Shanghai, China), according to the manufacturer's protocol. The concentration of four proteins was 8 µg mL^−1^ and the inhibitor celastrol was present at concentrations ranging from 0.5 to 8 µg mL^−1^.

### 2.18 Biochemical Parameters Assay

The WT, Δ*rocA*, and Δ::*rocA* were cultured to the logarithmic phase and were washed with PBS three times and adjusted to an OD_600 nm_ of 0.5. Then, celastrol or glyoxylate was added into PBS containing WT to final concentrations of 2 or 512 µg mL^−1^, respectively. After incubation in a shaker (160 rpm, 37 °C) for 4 h, the cells were collected by centrifugation (5500 rpm, 10 min) for use in subsequent assays.

#### 2.18.1 ROS Determination

The ROS accumulation of bacteria was measured with fluorescence probe DCFH‐DA (10 µmol L^−1^) using a ROS Assay kit (S0033S, Beyotime, Shanghai, China) as per the manufacturer's protocol. After incubation for 20 min, the fluorescence value was determined using a SpectraMax iD3 Microplate Reader (Molecular Devices), with the excitation wavelength at 488 nm and emission wavelength at 528 nm.^[^ ^25^ ^]^

#### 2.18.2 ATP Determination

Intracellular ATP levels of cells were determined using an Enhanced ATP Assay Kit (S0027, Beyotime, Shanghai, China) according to the manufacturer's instructions. Briefly, after the bacteria were collected by centrifugation, the bacterial precipitates were lysed by ATP lysate for 10 min to release the intracellular ATP, then the solution was centrifuged (12 000 rpm, 10 min, and 37 °C) and the supernatants were obtained for the subsequent measurement of ATP levels.^[^ ^33^ ^]^

#### 2.18.3 NAD^+^/NADH Determination

Cells were collected and suspended in 0.5 mL alkaline extracts. After ultrasonication, the NAD^+^ and NADH content were measured using the NAD^+^/NADH Assay Kit with WST‐8 (S0175, Beyotime, Shanghai, China) according to the manufacturer's instructions.

#### 2.18.4 NADP^+^/NADPH Determination

The NADP^+^ and NADPH in the bacteria were extracted with acidic and alkaline extracts. After centrifuging at 12 000 rpm for 10 min, the supernatant was collected and the NADP^+^ and NADPH content was measured using a NADP^+^/NADPH Assay Kit with WST‐8 (S0179, Beyotime, Shanghai, China) following the manufacturer instructions.

#### 2.18.5 Total Antioxidant Capacity Determination

Cells were collected by centrifugation (4500 rpm, 10 min) and suspended in an extract solution from a Micro total antioxidant capacity (T‐AOC) Assay Kit (A015‐3‐1, NJJCBIO, Nanjing, China). After ultrasonication, the cells were centrifuged (10 000 rpm, 10 min), and total antioxidant activity was measured according to the manufacturer's instructions.

#### 2.18.6 Determination of Glu and Asp Content

The Glu and Asp content was measured using the Glu Assay Kit (BC1580, Solarbio, Beijing, China) and Bacteria Asp ELISA Kit (YJ896471, mlbio, Shanghai, China), respectively, in line with the procedures stipulated by the manufacturers.

#### 2.18.7 Intracellular P5C Determination

The intracellular P5C content was determined by monitoring the amount of the P5C‐2‐aminobenzaldehyde complex as described by Li et al.^[^ ^11^ ^]^ Briefly, the cell was extracted in 3% (w/v) aqueous 5‐sulphosalicylic acid. Before 2‐aminobenzaldehyde (2‐AB) was added, the cell extract was boiled in a water bath for 10 min and added with trichloroacetic acid. The mixture was incubated at 37 °C for 1 h. It was centrifuged at 11 000 rpm for 10 min. The absorbance of the supernatant was measured at 443 nm. The P5C centration was calculated according to Beer–Lambert law, *A* = *Kbc*.

#### 2.18.8 Pro Determination

The intracellular concentration of Pro was determined using Proline Assay Kit (BC0295, Solarbio, Beijing, China) according to the manufacturer's instructions. Briefly, the cells were harvested and extracted in 5 mL 3% (w/v) aqueous 5‐sulphosalicylic acid. After ultrasonication (20 min) and boiling (10 min), the cell extract was reacted with glacial acetic acid and ninhydrin. The absorbance of the mixture was measured at 520 nm.

### 2.19 Protein Metabolism Assay

The WT, Δ*rocA*, and Δ::*rocA* were cultured to logarithmic phase, and WT cells were treated with 2 µg mL^−1^ celastrol or 512 µg mL^−1^ glyoxylate. After being incubated in a shaker (160 rpm, 37 °C) for 4 h, the bacterial cells were washed with PBS twice and obtained by centrifugation (5500 rpm, 10 min). The absorbance at OD_600 nm_ of the bacterial suspension of all groups was standardized to 0.8 in PBS. The same volume of bacterial suspension was centrifuged (5500 rpm, 10 min) and resuspended in 3 mL PBS. After ultrasonication, the protein content was determined with BCA Protein Assay Kit (MA0082, Meilunbio, Dalian, China). In addition, the proteins were observed with the help of 10% SDS‐PAGE.^[^ ^34^ ^]^

### 2.20 DNA Content Determination

The collection of bacteria was conducted in the same manner as the protein metabolism assay. After being resuspended in sterile PBS, the DAPI (Solarbio, Beijing, China) was added to bacterial suspensions. The fluorescence value was then determined using a microplate reader, with an excitation and emission wavelength of 340 and 488 nm, respectively.^[^ ^35^ ^]^

### 2.21 TUNEL Assay

For the TUNEL assay, cells of WT, Δ*rocA*, and Δ::*rocA* were grown to an OD_600 nm_ of 0.5, and WT cells were treated with 2 µg mL^−1^ celastrol or 512 µg mL^−1^ glyoxylate for 4 h. The cells were fixed and labeled using the one‐step TUNEL apoptosis assay kit (C1086, Beyotime Biotechnology, China). After the labeling reaction was stopped, the cells were washed three times with PBS and observed using a confocal laser scanning microscope (CLSM).

### 2.22 DAPI Staining Assay

Cells were grown to an OD_600 nm_ of 0.5, and WT cells were treated with 2 µg mL^−1^ celastrol or 512 µg mL^−1^ glyoxylate for 4 h. The cells were incubated with 0.02% Triton X‐100 and then fixed in 4% paraformaldehyde at 20 °C for 10 min. Finally, bacteria were stained with 10 µg mL^−1^ DAPI in the dark for 10 min and washed three times with PBS. The cells were observed under CLSM and a super‐resolution fluorescence microscope (SRFM).^[^ ^36^ ^]^

### 2.23 Statistical Analyses

Statistical analysis was performed using GraphPad Prism 7.0 software (GraphPad Software, San Diego, CA, USA). All data from at least three biological replicates were presented as means ± standard deviation (SD). Unless otherwise noted, unpaired two‐tailed Student's *t*‐test between two groups or one‐way analysis of variance among multiple groups were used to calculate *p*‐values (NS, not significant; * *p* < 0.05; ** *p* < 0.01).

## 3 Results

### 3.1 Celastrol Displays Potent Activity Against MRSA In Vitro

**Figure 2:**
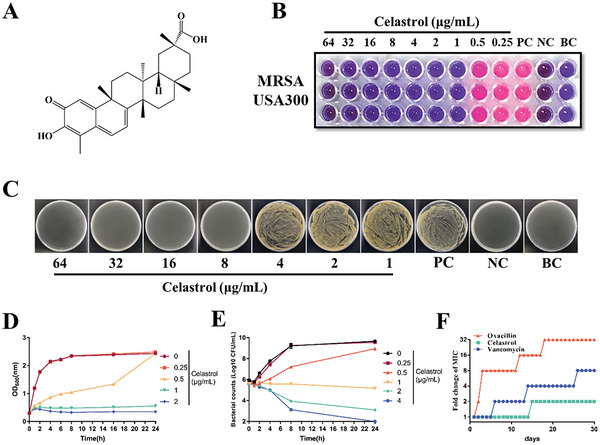
Anti‐MRSA activity of celastrol in vitro. A) Chemical structure of celastrol. B) MIC of celastrol against MRSA USA300. PC: positive control (MH containing MRSA USA300 without celastrol); NC: negative control (MH containing celastrol without MRSA USA300); BC: blank control (MH only). C) MBC of celastrol against MRSA USA300. D) Growth curve of celastrol against MRSA USA300. E) Time‐kill curve of celastrol against MRSA USA300. F) Resistance development of MRSA USA300 to celastrol, vancomycin, and oxacillin. Values indicate fold changes (in log2) in MIC relative to the MIC of the first passage.

The chemical structure of celastrol is shown in **Figure** **2**A. To evaluate its antibacterial activity in vitro, we determined its minimal inhibitory concentration (MIC) against a variety of bacteria, including Gram‐negative and Gram‐positive bacteria. Celastrol showed distinguished antibacterial activity against Gram‐positive bacteria, especially *S. aureus* with MICs ranging from 0.5 to 4 µg mL^−1^ (Figure 2B, **Table** **1**), compared to its effect on Gram‐negative bacteria. However, the MICs for Gram‐negative bacteria including *Escherichia coli* (*E. coli*), *Acinetobacter baumannii* (*A. baumannii*), and *Klebsiella pneumoniae* (*K. pneumoniae*), were more than 256 µg mL^−1^. The MBC of celastrol against MRSA USA300 was 8 µg mL^−1^ (Figure 2C).

**Table 1:** The activity of celastrol against pathogenic bacteria

Microorganism	MIC^a)^, ^b)^ [µg mL^−1^[	MIC^b)^, ^c)^ [µg mL^−1^[
G^+^ bacteria		
Methicillin‐resistant *S. aureus* (MRSA)		
*S. aureus* ATCC^a)^ BAA‐1717 (USA300)	1	1
*S. aureus* ATCC 43300	4	1
*S. aureus* 5ZB12 (clinical strain)	1	1
*S. aureus* 2ZG3 (clinical strain)	1	1
*S. aureus* 5ZB14 (clinical strain)	1	1
*S. aureus* 25FS35 (clinical strain)	1	1
*S. aureus* HX86 (clinical strain)	1	1
*S. aureus* YFC28 (clinical strain)	0.5	1
*S. aureus* HB119 (clinical strain)	4	1
*S. aureus* 25FS24 (clinical strain)	0.5	1
*S. aureus* 26FS31 (clinical strain)	2	0.5
*S. aureus* 6Y2C (clinical strain)	1	2
*S. aureus* 7SX2 (clinical strain)	1	1
*S. aureus* (MSSA)		
*S. aureus* ATCC 25923	2	1
*S. aureus* ATCC 29213	2	1
*Staphylococcus xylosus (S. xylosus)*		
*S. xylosus* ATCC 29971	2	4
*S. xylosus* ATCC 700404	4	1
*Streptococcus suis (S. suis)*		
*S. suis* ATCC 700794	16	<0.25
*S. suis* D2 (clinical strain)	32	2
*S. suis* D29 (clinical strain)	32	1
*S. suis* F2‐4 (clinical strain)	8	0.25
*S. suis* F8‐3 (clinical strain)	16	0.25
G^−^ bacteria		
*E. coli* ATCC 25922	>256	256
*E. coli* FS24 (clinical strain)	>256	256
*A. baumannii* B136 (clinical strain)	>256	>256
*A. baumannii* 3F2‐AB3 (clinical strain)	>256	256
*K. pneumoniae* E‐4C2 (clinical strain)	>256	256
*K. pneumoniae* E‐4H3 (clinical strain)	>256	256

^a)^ ATCC: American Type Culture Collection

^b)^ MIC^a^: the MICs of celastrol against pathogenic bacteria

^c)^ MIC^b^: the MICs of vancomycin against pathogenic bacteria.

We further tested the growth inhibitory effects of celastrol against MRSA USA300 (ATCC BAA‐1717). The growth curve showed that 0.25 µg mL^−1^ celastrol did not influence the growth of MRSA USA300 (Figure 2D). However, at a concentration of 0.5 µg mL^−1^, celastrol prolonged the logarithmic phase, increasing the time taken for bacteria to reach the stationary phase, and at 1 and 2 µg mL^−1^, it completely inhibited the growth of the strain.

Additionally, we monitored the bacteria viability of MRSA USA300 exposed to various concentrations of celastrol at different times (Figure 2E). No concentration could kill bacteria within 8 h, and only a concentration of 4 MIC (4 µg mL^−1^) celastrol could kill most bacteria over an extended period of time. Combined with the MBC, we confirm that celastrol is a bacteriostatic drug based on its MBC and MIC values.^[^ ^37^ ^]^

To evaluate celastrol resistance development, we performed an in vitro study in which MRSA USA300 was continuously exposed to a 1/2 MIC concentration of celastrol for 30 days. Unlike most antimicrobials, where resistance to antibiotics always occurs, the anti‐MRSA activity of celastrol merely decreased twofold. Compared to the 32‐fold and eightfold decrease in anti‐MRSA activity exhibited by oxacillin and vancomycin, respectively, celastrol displayed low levels of resistance development (Figure 2F). These results suggested that celastrol is a promising anti‐MRSA lead.

### 3.2 Celastrol Shows Potential Therapeutic Capabilities in Different Infection Models

**Figure 3:**
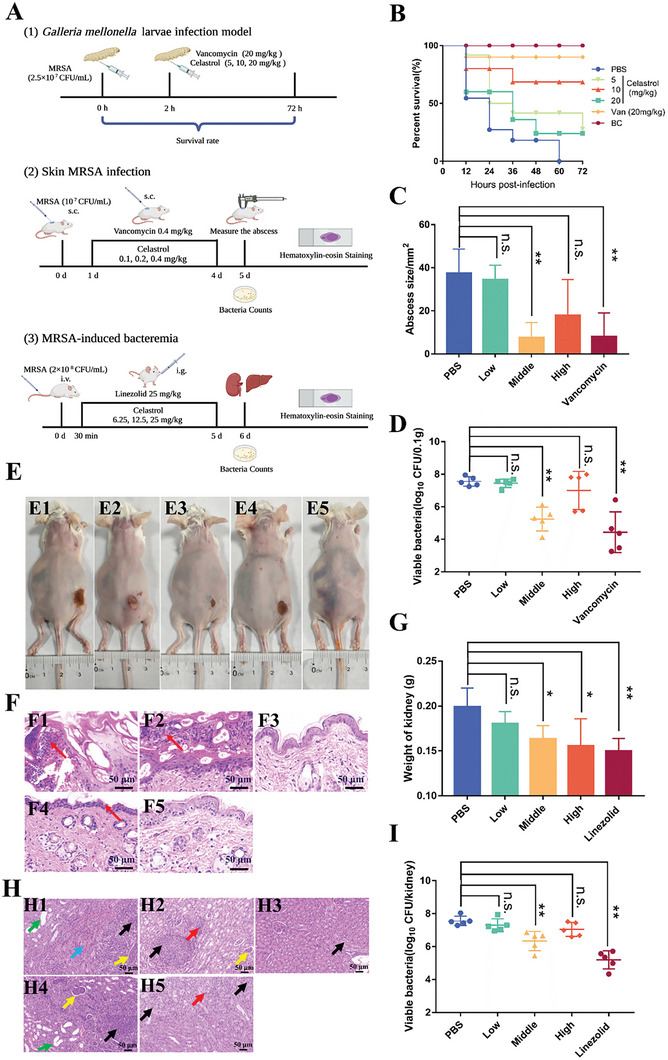
Anti‐MRSA activity of celastrol in vivo. A) Scheme of experimental protocol for *G. mellonella* infection, skin infection, and MRSA‐induced bacteremia models. Created with BioRender.com. B) Survival rates of *G. mellonella* larvae infected with MRSA USA300. Van: vancomycin, as a positive control; BC (blank control): only injected PBS without infections. C) Wound sizes in skin infection model after infection of MRSA USA300 in different groups. D) Bacterial burdens in wounds infected with MRSA USA300 in different groups. E) Image of wounds of mice infected with MRSA USA300 after 5 d of infection. E1: PBS treatment group; E2: low dose celastrol (0.1 mg kg^−1^) treatment group; E3: middle dose celastrol (0.2 mg kg^−1^) treatment group; E4: high dose celastrol (0.4 mg kg^−1^) treatment group; E5: vancomycin (0.4 mg kg^−1^) treatment group, as positive control. F) Histological evaluation of infected skin tissues of different groups. F1: PBS treatment group; F2: low dose celastrol (0.1 mg kg^−1^) treatment group; F3: middle dose celastrol (0.2 mg kg^−1^) treatment group; F4: high dose celastrol (0.4 mg kg^−1^) treatment group; and F5: vancomycin (0.4 mg kg^−1^) treatment group. G) Weight of kidneys of different groups. H) Histological evaluation of kidneys of different groups. H1: PBS treatment group; H2: low dose celastrol (6.25 mg kg^−1^) treatment group; H3: middle dose celastrol (12.5 mg kg^−1^) treatment group; H4: high dose celastrol (25 mg kg^−1^) treatment group; H5: linezolid (25 mg kg^−1^) treatment group, as a positive control. I) Bacterial burdens in the kidneys of different groups. NS, not significant; **p < 0.01 or *p < 0.05, compared with the PBS treatment group.

The encouraging antibacterial activity of celastrol and the difficulty with which MRSA develops drug resistance to it in vitro inspired us to further investigate its therapeutic potential in vivo. Three infection models including a *G. mellonella* larvae model and two mouse models of infection were utilized to test its efficacy (**Figure** **3**A). First, in the *G. mellonella* larvae assay, after infections with MRSA USA300 without drug treatment 90% of larvae died within 48 h. However, after treatment with 10 mg kg^−1^ celastrol or 20 mg kg^−1^ vancomycin, the survival rate of *G. mellonella* larvae was significantly improved, indicating that celastrol exhibited a striking protective effect on *G. mellonella* larvae from infections, and 5 or 20 mg kg^−1^ celastrol slightly improved the survival rate of *G. mellonella* larvae infected by MRSA USA300 (Figure 3B).

Skin infection is one of the diseases frequently caused by MRSA in hospitals.^[^ ^24^ ^]^ To evaluate the therapeutic potential of celastrol, we utilized a skin infection model. Abscesses of ≈40 mm^2^ were observed in the PBS treatment group (Figure 3C,E1). However, the size of the abscesses was significantly reduced after treatment with 0.2 mg kg^−1^ celastrol or 0.4 mg kg^−1^ vancomycin (Figure 3C,E3 and Figure 2E5). The results of histological examinations and bacteria counts displayed comparable trends (Figure 3D,F). In celastrol (0.2 mg kg^−1^) and vancomycin groups, only a few inflammatory cells were observed (Figure 3F3,F5) and the number of bacteria at the infectious sites decreased by more than 100 times, compared with the PBS treatment group. Notably, 0.1 and 0.4 mg kg^−1^ celastrol showed no therapeutic effect. These results suggested that celastrol could be used to treat MRSA skin infections in mice.

An MRSA‐induced bacteremia model was used to test the therapeutic effect of celastrol on severe MRSA diseases. Since celastrol is highly toxic and is not easy to be absorbed through injection,^[^ ^38^ ^]^ oral administration is preferred. The results showed that kidney weight significantly increased after the infection due to hyperemia. However, this condition improved after treatment with celastrol or linezolid (Figure 3G). The bacterial count assay suggested that a dosage of 12.5 mg kg^−1^ celastrol effectively reduced viable bacteria in the renal tissue (Figure 3I). The PBS treatment group showed surface abscesses (Figure S1, Supporting Information) as well as extensive renal tubular necrosis (both in the cortex and medulla) with unclear structures (Figure 3H1). A large number of granulocyte‐based inflammatory cell infiltration (Figure 3H1, black arrows), necrosis, and inflammatory cell clusters were also seen in the renal tubules (Figure 3H1 yellow arrows). Furthermore, tubular dilation (Figure 3H1, green arrow) and focal hemorrhage (Figure 3H1, blue arrow) were found in the PBS group. However, in the 12.5 mg kg^−1^ celastrol group, we only observed a small amount of renal tubular epithelial cell necrosis with hyperchromatic or fragmented pyknosis (Figure 3H3, black arrows). Additionally, we further investigated the effects of celastrol on organ damage. The results showed that dosages of 6.25 or 12.5 mg kg^−1^ celastrol did not result in any obvious pathological abnormalities to major organs, but the 25 mg kg^−1^ celastrol group showed certain toxicity to the liver and spleen, resulting in the apoptosis of lymphocytes and necrosis of liver cells (Figure S2, Supporting Information). Overall, celastrol has a therapeutic effect on MRSA bacteremia.

### 3.3 Discovery of a Novel Anti‐MRSA Target From Multi‐Omics Analysis

**Figure 4:**
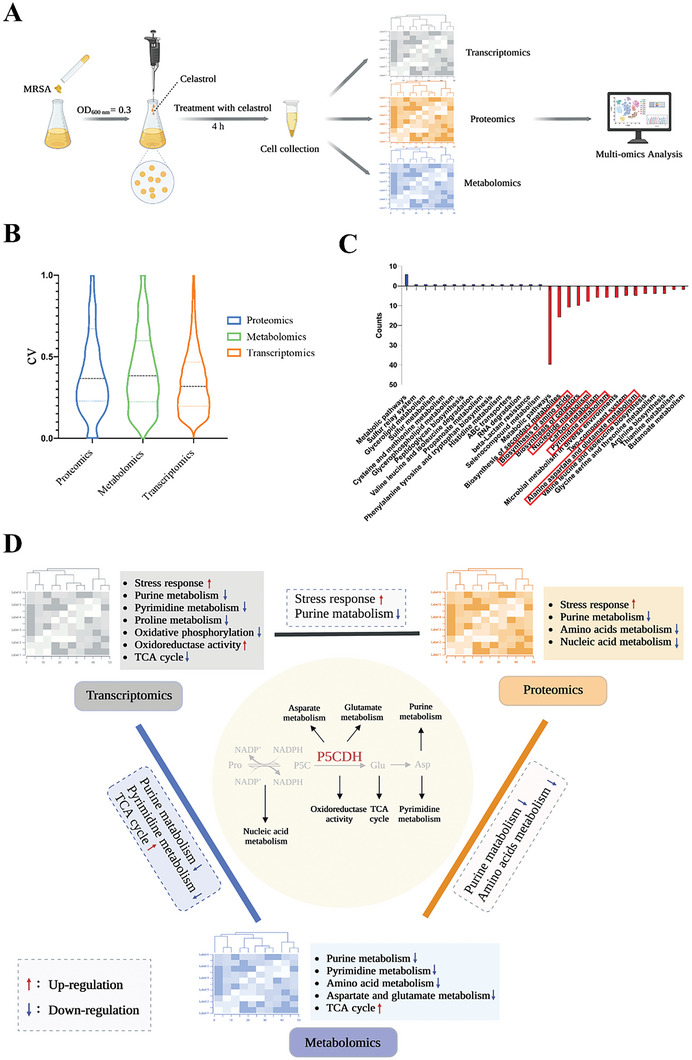
Multi‐omics analysis of MRSA USA300 treated with celastrol or DMSO. A) Scheme illustration of experimental protocol for multi‐omics. B) Violin plot. CV, coefficient of variation in different omics. C) KEGG enrichment analysis of multi‐omics pathways. D) P5CDH, as a potential anti‐MRSA target, was deduced from the multi‐omics analysis. Created with BioRender.com.

To reveal the possible target of celastrol in anti‐MRSA activity, the cellular changes were analyzed in MRSA USA300 after celastrol treatment using transcriptomics, proteomics, and metabolomics (**Figure** **4**A).

From the transcriptomic analysis, a total of 1292 differentially expressed genes (DEGs) (615 upregulated and 677 downregulated) with extremely significant (FC < 0.5 or >2, and *p* < 0.05) expression patterns before and after celastrol treatment were identified (Figure S3A, Supporting Information). KEGG and GO analysis showed virulence‐related pathways (two‐component systems (TCS), quorum‐sensing (QS) system, and carotenoid biosynthesis) and energy metabolism pathways (TCA cycle, oxidative phosphorylation, ABC transporters, and glycolysis) were downregulated, whereas DNA replication and oxidoreductase activity were upregulated (Figure S3, Supporting Information). Taken together, these upregulated DEGs are largely associated with the metabolism and degradation of secondary substances, whereas the processes of carbon source metabolism and nitrogen source metabolism were significantly inhibited.

Contrary to the results of the transcriptomics analysis, proteomics analysis showed the pathways including ABC transporters and QS system were upregulated after celastrol treatment. Overall, celastrol significantly upregulated the stress response and ion channel activation processes, whereas downregulating nucleic acid metabolism, primary substance metabolism, cell activity, purine metabolism, and DNA repair (Figure S4, Supporting Information).

There were 30 different metabolites between the control and drug groups in metabolomics data (Figure S5, Supporting Information). KEGG pathway analysis revealed that the TCA cycle and biosynthesis of cofactors were significantly downregulated. In addition, molecules related to purine and pyrimidine metabolism, as well as the metabolism of histidine, alanine, aspartate, glutamate, and arginine were significantly downregulated after celastrol treatment.

Subsequently, tri‐omics data were further analyzed using pathway analysis across omics (Figure 4B). In the combined transcriptomics‐proteomics analysis, nitrogen metabolism, amino acid synthesis, and stress response showed a strong correlation. Similarly, carbon metabolism, nitrogen metabolism, and energy metabolism were significantly downregulated in the combined transcriptomics‐metabolomics analysis. After KEGG enrichment analysis of the significantly downregulated proteins and metabolites simultaneously, we found purine metabolism and pyrimidine metabolism were significantly downregulated. Finally, multi‐omics analysis demonstrated that celastrol upregulated stress response and oxidative phosphorylation, whereas downregulated biosynthesis of amino acids and nucleotide, aspartate, glutamate, and pyrimidine metabolisms. Nucleotides, purine, and pyrimidine are essential for DNA synthesis. Therefore, we reasoned that celastrol inhibited cell growth via oxidative stress and the inhibition of DNA synthesis. Additionally, these results indicated an anti‐MRSA role of celastrol through multiple pathways (Figure 4C).

According to multi‐omics analysis, we speculated that the antibacterial activity of celastrol was related to multiple pathways including oxidative stress and energy metabolism. Among them, downregulated purine and pyrimidine metabolisms were observed in all tri‐omics data. Therefore, we hypothesized that an important antibacterial target of celastrol against MRSA could influence the synthesis of purine and pyrimidine. Asp, the common precursor of the purine ring and pyrimidine ring, was also downregulated in metabolomics data after celastrol treatment, which suggested that the pathway related to Asp metabolism needs to be further examined and analyzed. The synthesis of Asp is related to the TCA cycle and Glu metabolomics, but both were downregulated. Notably, Pro, the major source of glutamate, showed no change in the metabolomics analysis. Thus, we preliminarily deduced that the target was involved in the Pro‐Glu reaction. PRODH and P5CDH are the two enzymes in this reaction, but P5CDH could affect electron transfer and the TCA cycle. Importantly, Halsey^[^ ^10^ ^]^ and Li et al.^[^ ^11^ ^]^ reported that P5CDH‐deficient strains of MRSA had significant growth yield defects whereas PRODH‐deficient strains did not. Therefore, P5CDH was speculated to be a target of celastrol (Figure 4D).

### 3.4 P5CDH is a Potential Antimicrobial Target

**Figure 5:**
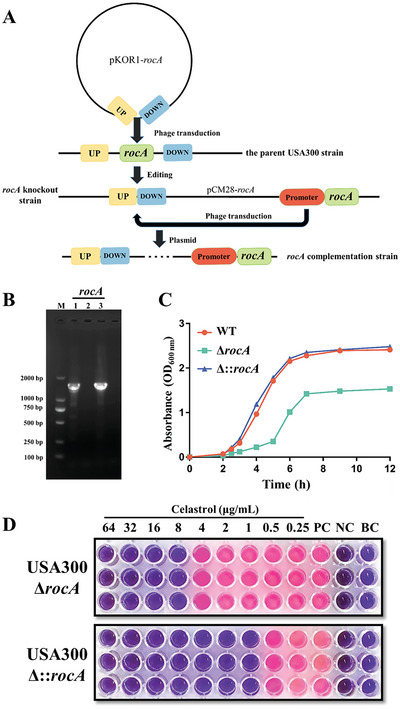
P5CDH is a potential antimicrobial target. A) Schematic model of construction of Δ*rocA* and Δ::*rocA*. B) PCR identification of Δ*rocA* and Δ::*rocA*. M: DL2000 DNA marker; 1: WT; 2: Δ*rocA*; 3: Δ::*rocA*. C) Growth curve of WT, Δ*rocA*, and Δ::*rocA*. D) MIC of celastrol against Δ*rocA* and Δ::*rocA*. PC: positive control (MH containing MRSA USA300 without celastrol); NC: negative control (MH containing celastrol without MRSA USA300); BC: blank control (MH only).

To further identify P5CDH as a potential antimicrobial target, we constructed an MRSA USA300 *rocA* knockout (Δ*rocA*) and complemented strain (Δ::*rocA*) using allelic replacement (**Figure** **5**A; the results of these processes are shown in Figure S6, Supporting Information) and confirmed by PCR (Figure 5B; Figure S6J, Supporting Information) and DNA sequencing. Based on the results of growth curves (Figure 5C), the deletion of *rocA* significantly impaired bacterial growth. Specifically, the Δ*rocA* exhibited a reduced growth rate and yield as compared to the wild‐type (WT). The complemented strain would then phenocopy the WT with regard to growth. Altogether, these results suggest that P5CDH is important for MRSA growth and is a potential antimicrobial target. In addition, after the mutation, the MIC of celastrol against MRSA changed from 1 to 8 µg mL^−1^, but it returned to 1 µg mL^−1^ after supplementing the *rocA* gene (Figure 5D). These results showed that the antibacterial activity of celastrol was partly realized by the inhibition of P5CDH.

### 3.5 P5CDH Binding by Celastrol

**Figure 6:**
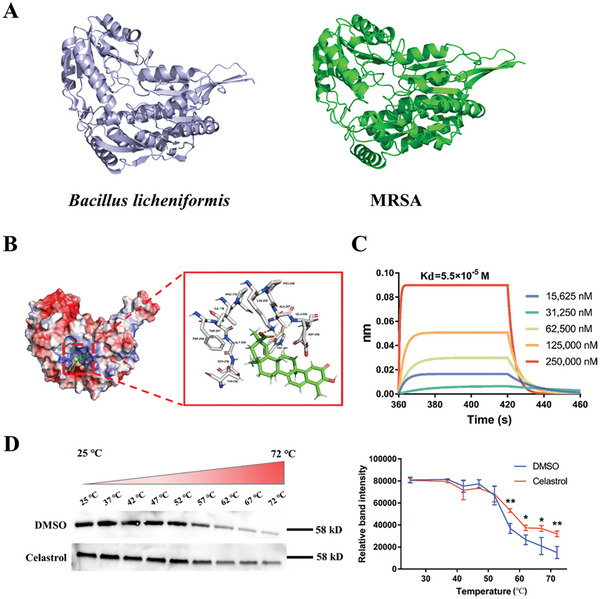
The site and binding affinity of celastrol to P5CDH. A) Homology model building of MRSA P5CDH (right) was performed according to the *B. licheniformis* P5CDH (left). B) Predicted binding mode of celastrol in P5CDH binding pocket obtained from molecular docking. C) Kinetic analysis by BLI of binding of celastrol to P5CDH. D) Cellular thermal shift assays to verify celastrol binding to P5CDH.

Since celastrol did not significantly affect the expression of P5CDH in the proteomics analysis, we speculated that celastrol exerts antibacterial activity by targeting P5CDH and affecting its function. Due to the poorly characterized 3D crystal structure profile of MRSA P5CDH, we searched the homology of sequence alignment of P5CDH residues in the Protein Data Bank (PDB) and selected the *Bacillus licheniformis* (*B. licheniformis*) P5CDH (3RJL) as the homology model, which shares a 59.9% sequence identity with that of MRSA P5CDH (**Figure** **6**A). The results of the PROCHECK, ERRAT, and Verify 3D programs are shown in Figure S7 (Supporting Information), which shows that the established 3D model of P5CDH was a reasonable structure and could be used for subsequent research. After molecular docking, we found that celastrol is able to bind to P5CDH and occupy the binding pocket. As shown in Figure 6B, celastrol adopted a conformation that formed an ionic interaction and a hydrogen bond with residues from the active sites Lys205 and Glu208, respectively.

To further demonstrate that celastrol can directly bind to P5CDH, we expressed the P5CDH protein (Figure S8, Supporting Information) and then measured their interaction. BLI suggested that celastrol could interact with P5CDH and the dissociation constant Kd had a value of 5.5 × 10^−5^ m (Figure 6C). Meanwhile, we found the interaction intensity was dose‐dependent when the concentration of celastrol was in the range of 15 625–250 000 nm. As further confirmation of binding, the intracellular binding of celastrol to the P5CDH protein was observed using CETSA, and results similar to those obtained from BLI were obtained. Celastrol was able to bind to P5CDH and make P5CDH thermally stable (Figure 6D). In summary, these results suggested that celastrol could bind to P5CDH.

### 3.6 K205A and E208A are Key Binding Sites of P5CDH to Celastrol

**Figure 7:**
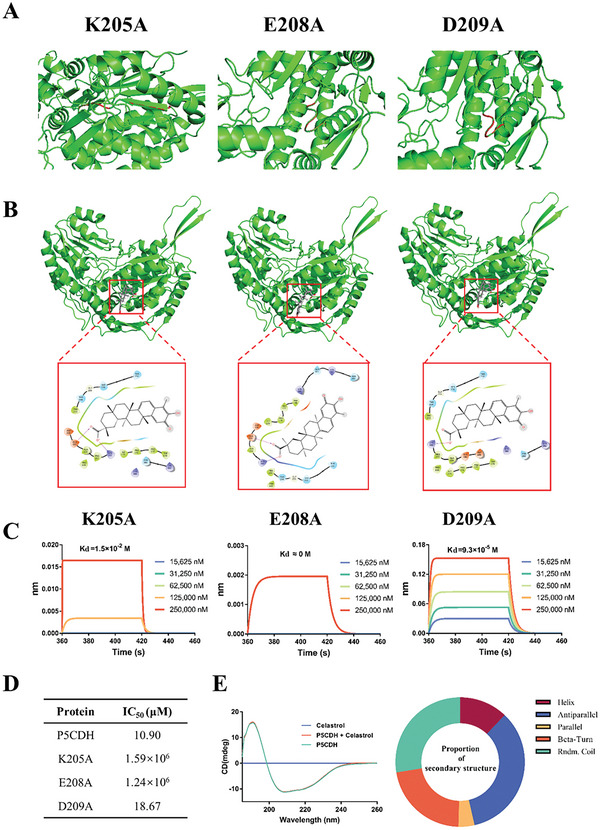
Key residues in the interaction between celastrol and P5CDH A) Position (red) of three residues (K205A, E208A, and D209A) in each protein. B) Structural models of three mutated proteins complexed with celastrol. C) Kinetic analysis by BLI of binding of celastrol to three mutated proteins. D) IC_50_ values of celastrol against P5CDH, K205A, E208A, and D209A. E) Normalized circular dichroism (CD) spectra of P5CDH in the presence and absence of celastrol.

To locate the docking site where celastrol binds to P5CDH, molecular docking, and site‐directed mutagenesis technologies were used in the subsequent study. According to the results of molecular docking, three binding sites were successfully predicted, specifically Lys205 (K205A), Glu208 (E208A), and Asp209 (D209A) (**Figure** **7**A,B). Thereafter, we constructed three mutant plasmids and expressed related proteins (the results of sequencing, expression, and purification of the mutant plasmids and proteins are shown in Figure S8, Supporting Information). We further analyzed the interaction between celastrol and these three proteins using BLI. The results showed that only the D209A protein retained its original level of activity after the mutation, and the Kd values of the other two mutant proteins decreased by more than 1000‐fold (Figure 7C). Subsequently, we performed an enzyme activity analysis test to verify these results. The resulting IC_50_ values show that celastrol had a little inhibitory effect on the enzyme activities of K205A and E208A. However, the IC_50_ value of D209A changed slightly which is ≈0.71‐fold compared to the native protein (Figure 7D). These results suggested that K205 and E208 were key residues for binding. Additionally, the circular dichroism (CD) curve of P5CDH showed no difference in the presence or absence of celastrol (Figure 7E), suggesting that celastrol did not affect the second structure of P5CDH.

### 3.7 Celastrol Promotes Oxidative Damage

**Figure 8:**
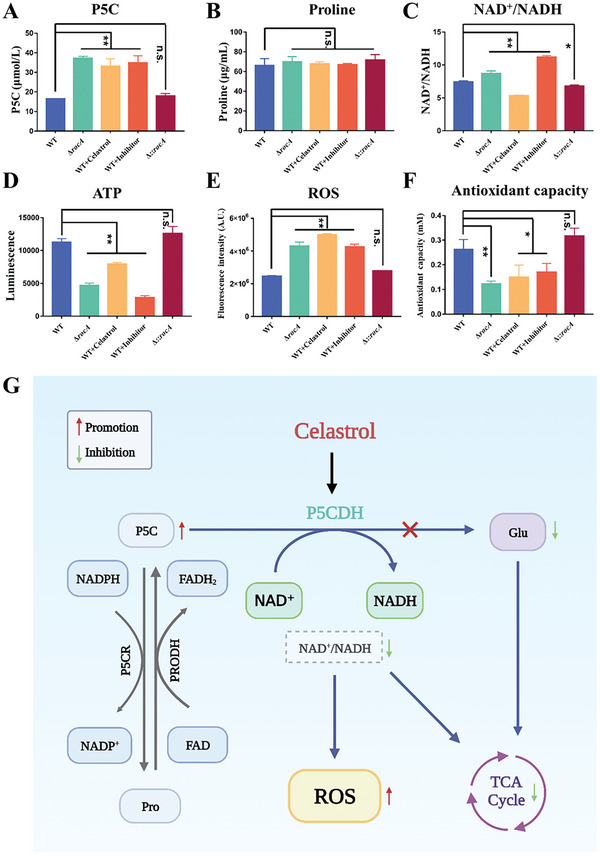
Celastrol promotes oxidative damage and induces cell death. A) P5C content. B) Pro content. C) Ratio of NAD^+^/NADH. D) Intracellular ATP level. E) ROS level. F) Antioxidant capacity. G) Schematic diagram of celastrol inducing oxidative damage. Created with BioRender.com. WT: wild‐type strain group; Δ*rocA*: *rocA* knockout strain group; celastrol: WT treated with the celastrol group; Inhibitor: WT treated with glyoxylate group; Δ::*rocA*: *rocA* complemented strain group. NS, not significant; **p < 0.01 or *p < 0.05, compared with the WT group.

Since P5CDH is essential to prevent the dense circulation of P5C‐Pro and to avoid the production of ROS due to electron loss,^[^ ^39^ ^]^ we speculated that celastrol could play an antibacterial role through oxidative damage. To that end, we first explored the changes in Pro and P5C content after treatment with celastrol. Meanwhile, the WT was treated with glyoxylate, which is the most potent P5CDH inhibitor reported to date with Ki = 0.27 mm and forms identical hydrogen bonds with P5CDH,^[^ ^40^ ^]^ as a positive control. Consistently, the P5C content significantly increased in celastrol or inhibitor treatment and the Δ*rocA* group, compared to the WT or the Δ::*rocA* group (**Figure** **8**A). However, celastrol did not change the Pro content, which was also verified by metabolomics data (Figure 8B). These results suggest that celastrol could disturb the balance of P5C‐Pro circulation. Meanwhile, the reaction catalyzed by P5CDH is accompanied by the conversion of NAD^+^ to NADH and electron transfer, which is related to the TCA cycle and oxidative stress. Thus, we tested the ratio of NAD^+^/NADH, ATP content, ROS level, and antioxidant capacity. The results showed that in the Δ*rocA* group and inhibitor treatment group, the ratio of NAD^+^/NADH significantly increased (Figure 8C) and the ATP level declined, suggesting that P5CDH affected the TCA cycle (Figure 8D). Interestingly, celastrol treatment reduced the ratio compared to the WT or the Δ::*rocA* group and increased the generation of ATP compared to the Δ*rocA* group. We reasoned that this phenomenon was because celastrol promoted the electron transfer chain and the TCA cycle. Due to the loss of electrons, the ROS level increased in Δ*rocA*, and celastrol or inhibitor treatment group. Among these groups, the increase of ROS was the most significant in the celastrol treatment group (Figure 8E), which may be because celastrol is a multi‐pathway antibacterial agent and can stimulate ROS generation in numerous ways.^[^ ^20^, ^21^, ^41^ ^]^ Meanwhile, the inhibition of P5CDH activity reduced antioxidant content, which lead to a decrease in antioxidant capacity (Figure 8F). *N*‐acetylcysteine (NAC), a ROS scavenger, changed the MIC of celastrol against MRSA USA300 from 1 to 64 µg mL^−1^ (Figure S9, Supporting Information), indicating that oxidative damage is an important antibacterial mechanism of celastrol (Figure 8G).

### 3.8 Celastrol Induces Cell Death

**Figure 9:**
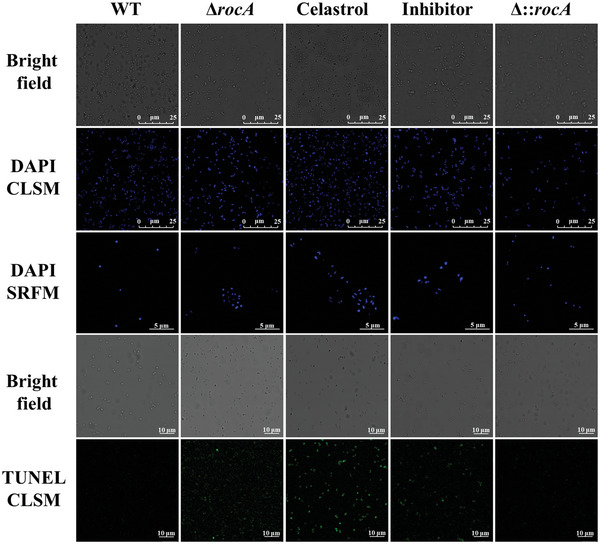
Detection of MRSA cell death under CLSM and SRFM. Bright field: cells were observed under bright field using CLSM; DAPI CLSM: cells stained with DAPI were observed by CLSM with excitation wavelength at 340 nm and emission wavelength at 488 nm; DAPI SRFM: cells stained with DAPI were observed by SRFM with excitation wavelength at 340 nm and emission wavelength at 488 nm; TUNEL CLSM: cells stained with TUNEL were observed by CLSM with excitation wavelength at 480 nm and emission wavelength at 520 nm. WT: wild‐type strain group; Δ*rocA*: *rocA* knockout strain group; celastrol: WT treated with celastrol group; Inhibitor: WT treated with glyoxylate group; Δ::*rocA*: *rocA* complemented strain group.

To study cell death in MRSA after celastrol treatment, DAPI was used to label DNA in coalescing cells. Chromosome condensation was observed in Δ*rocA*, celastrol, or inhibitor treatment groups, indicating the occurrence of cell death. Consistently, cells of WT and Δ::*rocA* groups appeared smooth and round, and the fluorescence intensity of cells was the same. However, the shape of cells and the fluorescence intensity underwent a change in the Δ*rocA*, celastrol, and inhibitor groups. In particular, the cells treated with celastrol showed morphological characteristics of cell death including apoptosome formation and shrinkage. In the TUNEL staining assay, compared to WT cells that did not undergo drug treatment, WT cells treated with celastrol showed intense fluorescence, suggesting the appearance of DNA double‐strand breaks. The slight fluorescence in the Δ*rocA* group and inhibitor treatment group showed that cell death had occurred in a smaller proportion of cells (**Figure** **9**).

### 3.9 Celastrol Inhibits DNA Synthesis

**Figure 10:**
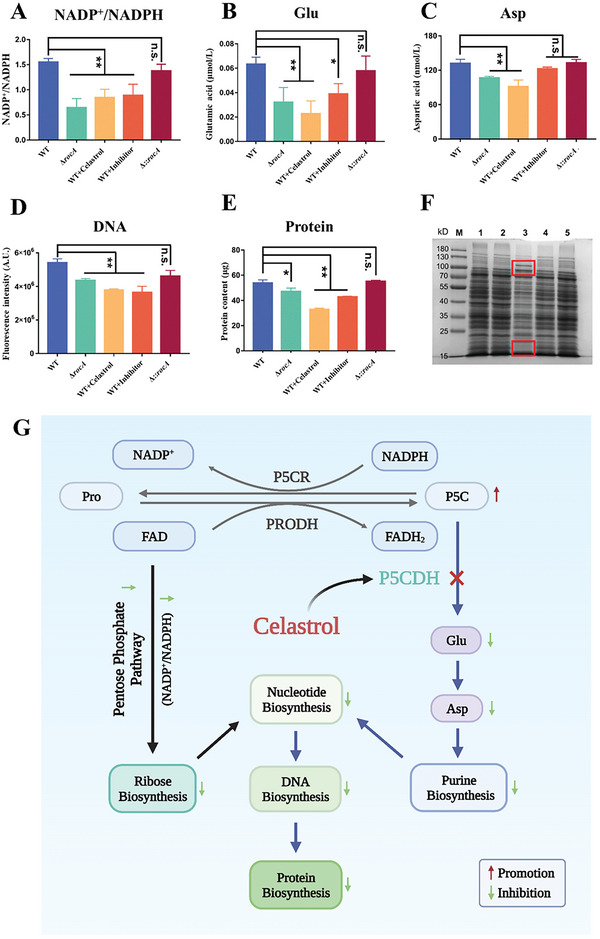
Celastrol inhibits DNA synthesis. A) Ratio of NADP^+^/NADPH. B) Glu content. C) Asp content. D) Fluorescence intensity of DNA. E) Protein content. F) SDS‐PAGE gel image. G) Schematic diagram of inhibition of synthesis of DNA and proteins by celastrol. Created with BioRender.com. M: maker; lanes 1: WT without treatment group; lanes 2: Δ*rocA* group; lanes 3: WT cells treated with celastrol group; lanes 4: WT cells treated with inhibitor group; lanes 5: Δ::*rocA* group. WT: wild‐type strain group; Δ*rocA*: *rocA* knockout strain group; celastrol: WT treated with celastrol group; Inhibitor: WT treated with glyoxylate group; Δ::*rocA*: *rocA* complemented strain group. NS, not significant; **p < 0.01 or *p < 0.05, compared with the WT group.

Considering that the Glu‐Pro reaction is accompanied by NADPH/NADP^+^ conversion, which is tightly coupled to the pentose phosphate pathway, we hypothesized that celastrol exerted anti‐MRSA activity likely through the inhibition of DNA synthesis. The decreased ratio of NADP^+^/NADPH in WT cells treated with celastrol or inhibitor and Δ*rocA* groups, compared to that of the WT or Δ::*rocA* group, suggests that the synthesis of ribose 5‐phosphate was inhibited (**Figure** **10**A). Glu is a central molecule in amino acid metabolism contributing to the synthesis of several amino acids and its *α*‐ group can be transferred to oxalic acid to form Asp. Asp is an important precursor for the synthesis of purines and pyrimidines. Therefore, the decrease of Glu and Asp (Figure 10B,C) limited the synthesis of DNA. Additionally, we consistently observed that the DNA content decreased (Figure 10D). Considering that DNA can affect the expression of proteins, we investigated the effect of celastrol on proteins. The results showed that protein contents of the Δ*rocA*, celastrol, or inhibitor treatment groups were significantly reduced, indicating that P5CDH could affect protein synthesis (Figure 10E). Interestingly, celastrol promoted the expression of proteins between 100–130 kD and inhibited the expression of proteins between 15 and 25 kD. However, the expression of proteins with different molecular weights had no significant effect in the Δ*rocA* group, compared with the WT group (Figure 10F). Overall, celastrol could combat MRSA via the inhibition of DNA synthesis (Figure 10G).

## 4 Discussion

Infectious diseases caused by MRSA are a matter of global concern due to the resulting high morbidity, mortality, and therapy costs in the clinic. The Centers for Disease Control and Prevention (CDC) has reported that more than 19 000 hospital deaths occur due to MRSA infections every year in the United States.^[^ ^42^ ^]^ Therefore, it is imperative to search for novel antimicrobial agents to combat MRSA.

Compared to conventional antibiotics, natural products have attracted greater attention due to their easily available sources, low costs, pharmacological activities, and minimal side effects.^[^ ^24^, ^43^ ^]^ A large number of natural products, including kuwanon G, *α*‐mangostin, and isobavachalcone have been recently reported to have excellent antibacterial effects on MRSA.^[^ ^25^, ^44^ ^]^ Among them, celastrol not only shows antibacterial activity but also mitigates staphyloxanthin biosynthesis and biofilm formation.^[^ ^45^ ^]^ In this study, celastrol exhibited the capacity against all MRSA strains tested in vitro; however, it showed no obvious activity against Gram‐negative bacteria. The selective activity of celastrol against bacteria was possibly due to the difference between P5CDH in Gram‐positive and Gram‐negative bacteria. P5CDH is a separate enzyme in Gram‐positive bacteria, whereas it is combined with Pro dehydrogenase into a single bifunctional enzyme known as Pro utilization A (PutA) in Gram‐negative bacteria.^[^ ^46^ ^]^ Besides, it may also be related to structural differences. Compared to Gram‐positive bacteria, Gram‐negative bacteria have an outer membrane, and it can block large molecules such as lysozyme, penicillin, and detergent from entering the bacteria. Therefore, celastrol may be blocked by the outer membrane of Gram‐negative bacteria and cannot enter the cells. Additionally, drug resistance is a thorny issue and bacteria are prone to developing resistance to current antibiotics. Next‐generation antibacterial agents need to diminish the development of antibiotic resistance.^[^ ^26^ ^]^ Celastrol displayed low levels of resistance development due to multiple pathways, highlighting its potential to combat MRSA.

Since the CDC reported that 64% of MRSA isolates were of the USA300 strain in infected patients in the USA, we choose USA300 as the test strain in this study.^[^ ^47^ ^]^ Notably, in three in vivo experiments, we found that only the medium dose of celastrol had a good therapeutic effect on the experimental targets, suggesting the therapeutic window of celastrol is very narrow, which may be related to its toxicity. Xu et al.^[^ ^48^ ^]^ reported that the LD_50_ value of celastrol intravenously in ICR mice is 3.12 mg kg^−1^. Other adverse effects include hepatotoxicity and renal injury,^[^ ^49^, ^50^, ^51^, ^52^, ^53^ ^]^ which is consistent with the results of our study. Moreover, we found that 25 mg kg^−1^ celastrol could induce apoptosis in spleen cells, which has not been reported previously. Importantly, celastrol has been found to have immunosuppressive effects,^[^ ^54^ ^]^ which may account for its low activity at high concentrations. These results indicated that celastrol may not be suitable for direct use as an anti‐MRSA drug, and should instead be considered as a lead compound for structural modification or preparations.

The multi‐omics analysis demonstrated that celastrol affected multiple metabolic pathways, including glycolysis, fatty acid metabolism, and the two‐component system. Among them, carotenoid biosynthesis related to staphyloxanthin was inhibited after celastrol treatment, which was consistent with the results of a recent study by Yehia et al.^[^ ^45^ ^]^ Celastrol has been reported to exhibit anti‐biofilm properties toward MRSA.^[^ ^45^ ^]^ Results of the multi‐omics analysis have led us to speculate this may be because celastrol can inhibit QS and TCS. QS is population density‐dependent and environment‐dependent gene regulation that occurs through cell‐cell communication^[^ ^55^ ^]^ and plays an important role in the process of infections.^[^ ^56^, ^57^ ^]^ TCSs are central pillars of environmental sensing, affecting the formation of biofilms and the expression of virulence factors.^[^ ^58^, ^59^ ^]^ In recent years, the development of anti‐QS and anti‐TCS drugs has been a popular direction to combat MRSA infections. Collectively, several pathways such as DNA repair, have been reported^[^ ^60^ ^]^ whereas other pathways have not been concerned. These provide potential approaches for developing anti‐MRSA drugs.

Proline metabolism is of vital importance for bacterial growth. Li et al.^[^ ^11^ ^]^ reported that the lag phase of deletion of the ycgN gene (the gene for P5CDH in *B. licheniformis*) in *B. licheniformis* was prolonged by 4–8 h compared to the WT. Accordingly, a similar situation was also observed in *S. aureus*.^[^ ^10^ ^]^ Therefore, we reasoned that P5CDH may be an antibacterial target. Three observations in this study provided evidence for P5CDH as the possible target of celastrol: I) the high binding affinity of celastrol to P5CDH in a dose‐dependent model; II) Celastrol improved thermal stability of P5CDH; III) celastrol inhibited the activity of P5CDH; and (IV) *rocA* deficiency impaired the growth of MRSA, and the antibacterial effect of celastrol on Δ*rocA* was weakened. CETSA is an experimental approach aimed at examining the binding of intracellular drugs to target proteins, based on the principle that target proteins become stable upon binding with drug molecules.^[^ ^61^ ^]^ The enhancement in thermal stability of P5CDH post celastrol treatment indicates successful binding of celastrol to P5CDH within the cells. Additionally, the results of CETSA, which monitors processes of off‐target effects,^[^ ^61^ ^]^ suggest that celastrol can bind to P5CDH in cells without off‐target effects. Moreover, the growth of bacteria without the *rocA* gene suggests that this gene is important for bacterial growth, but not essential. Meanwhile, compared with the WT, the MIC of celastrol against Δ*rocA* only increased to 8 µg mL^−1^, indicating that celastrol combats MRSA through multiple pathways. Additionally, we found two key sites (K205 and E208) on P5CDH to which celastrol binds. Since Lys is an alkaline amino acid and the functional group that interacts with celastrol is acidic, they form strong chemical bonds.^[^ ^62^ ^]^ According to the position and rotation of celastrol, E208, and C=O can result in an interaction (hydrogen bond).

Given that glyoxylate is an inhibitor of P5CDH,^[^ ^40^ ^]^ we used it as the control in mechanistic studies. The upstream and autocatalytic reactions of P5CDH are accompanied by the electron transfer of coenzyme II and coenzyme I, respectively. Disturbing the balance of P5C‐Pro circulation and blocking the P5CDH‐catalyzed reaction would impact electron transfer. The lack of P5CDH enhances P5C accumulation, whereas it did not affect Pro content, which induced ROS increase.^[^ ^11^, ^63^ ^]^ ROS‐mediated oxidative damage has been widely recognized as a common pathway for antibiotic killing.^[^ ^64^ ^]^ Celastrol has been reported to induce ROS generation in a variety of ways.^[^ ^20^, ^21^, ^41^ ^]^ Consistently, more ROS are detected in MRSA after celastrol treatment, compared with other groups, suggesting that celastrol induces ROS production through other pathways apart from P5CDH. Treatment with the ROS scavenger NAC partly weakened the antibacterial activity of celastrol, indicating that ROS induced by celastrol inhibited the growth of MRSA. Consistently, celastrol has been reported to increase intracellular ROS accumulation, and NAC can block the process induced by celastrol.^[^ ^65^ ^]^ Additionally, the ratio of NAD^+^/NADH reflected the change in the TCA cycle.^[^ ^66^ ^]^ We confirmed that celastrol could inhibit the TCA cycle and decrease ATP generation, which may serve as another anti‐MRSA mechanism of celastrol. Numerous small‐molecule compounds that intervene in bacterial redox balance, which reduces ATP generation and enhances ROS generation,^[^ ^67^, ^68^, ^69^ ^]^ have been identified. Moreover, a study by Dwyer et al.^[^ ^70^ ^]^ found that bacteria, like eukaryotic cells, possess mechanisms of apoptotic‐like death (ALD) under specific stimulation, such as platelets, beetroot extract, and chitooligosaccharides.^[^ ^71^, ^72^, ^73^ ^]^ Meanwhile, P5CDH plays an important role in cell death.^[^ ^74^ ^]^ Interestingly, our study revealed that the inhibition of P5CDH activity by celastrol would induce ALD through the accumulation of P5C, which may subsequently lead to cell death. This evidence provides a mechanistic explanation for its antibacterial activity. In addition to causing oxidative damage and cell death in MRSA, we found that celastrol can inhibit DNA synthesis. Inhibition of DNA synthesis is a mainstream antibacterial mechanism.^[^ ^75^ ^]^ Ribose and purine are important precursors of nucleotides, which are the basic units of DNA. Therefore, celastrol affects DNA synthesis by affecting the production of ribose and purine.^[^ ^76^, ^77^ ^]^

## 5 Conclusion 

In conclusion, our results have shown that celastrol exhibits robust antibacterial activities against standard and clinical strains of MRSA, and displays low levels of resistance development. The mechanism of this antibacterial activity is proposed to be based on competitive binding to P5CDH, which activates multiple pathways. Celastrol is a promising candidate for the development of novel antibiotic agents to combat MRSA‐associated infections.

## Conflict of Interest

Shuguang Yuan is the cofounder of AlphaMol Science Ltd.

## Author Contributions

Y.L., Z.Y., and Q.Q. designed the experiments; Z.Y., Z.Z., M.Z., C.S., Y.D., X.H., C.D., and Y.Z. performed the experiments; M.X. and H.P. conducted the molecular docking; Z.Y. and J.W. drew the pictures; and Z.Y. wrote the paper. Q.Q., S.Y., and Y.L. revised the paper.

## Supporting information

Supporting Information

## Data Availability

The data that support the findings of this study are available from the corresponding author upon reasonable request. Additionally, raw omics data can be viewed in NODE (https://www.biosino.org/node) by pasting the accession OEP003793 into the text search box.
