# Supplementary material for: Celastrol Combats Methicillin‐Resistant Staphylococcus aureus by Targeting Δ1‐Pyrroline‐5‐Carboxylate Dehydrogenase
Source: Adv Sci (Weinh). 2023 Jun 28;10(25):2302459. doi: 10.1002/advs.202302459 (PMC10477891; doi:10.1002/advs.202302459)
Supplement: Supplementary file 1 — Supporting Information [file ADVS-10-2302459-s001.pdf]

ADVANCED SCIENCE Open Access

Supporting Information

for *Adv. Sci.*, DOI 10.1002/advs.202302459

Celastrol Combats Methicillin-Resistant *Staphylococcus aureus* by Targeting Δ^1-Pyrroline-5-Carboxylate Dehydrogenase

Zhongwei Yuan, Jun Wang, Qianwei Qu, Zhenxin Zhu, Marc Xu, Mengmeng Zhao, Chongxiang Sun, Haixin Peng, Xingyu Huang, Yue Dong, Chunliu Dong, Yadan Zheng, Shuguang Yuan and Yanhua Li**

Supporting Information

Celastrol combats methicillin-resistant *Staphylococcus aureus* by targeting Δ^1-pyrroline-5-carboxylate dehydrogenase

Zhongwei Yuan[1], Jun Wang[1], Qianwei Qu[1], Zhenxin Zhu[1], Marc Xu[2], Mengmeng Zhao[1], Chongxiang Sun[1], Haixin Peng[1], Xingyu Huang[1], Yue Dong[1], Chunliu Dong[1], Yadan Zheng[1], Shuguang Yuan[2,*], and Yanhua Li[1,*]

[1]Heilongjiang Key Laboratory for Animal Disease Control and Pharmaceutical Development, College of Veterinary Medicine, Northeast Agricultural University, Harbin 150030, China.

[2]Shenzhen Institutes of Advanced Technology, Chinese Academy of Sciences, Shenzhen 518055, China.

***Corresponding Authors**

E-mail: liyanhua@neau.edu.cn, shuguang.yuan@siat.ac.cn

Supplementary Figures

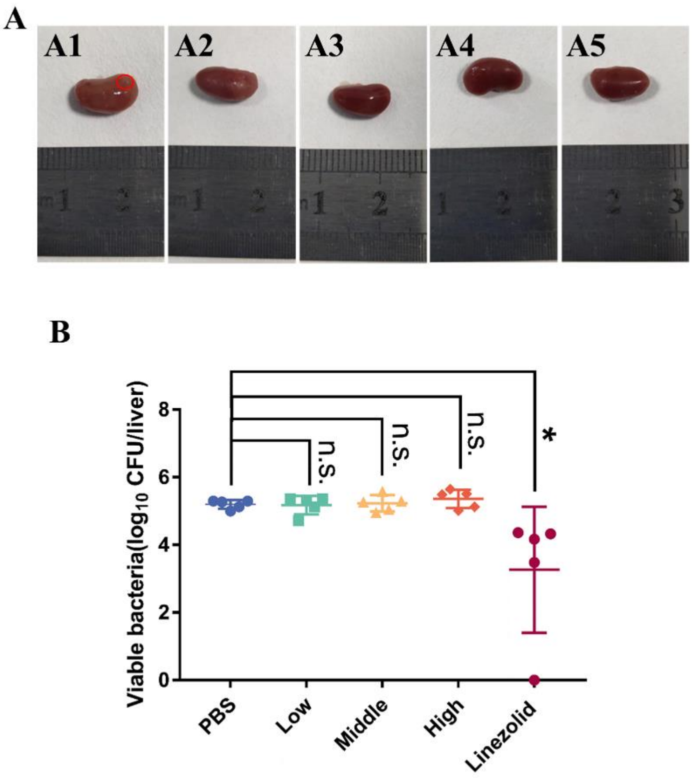

Figure S1. Therapeutic effect of celastrol on bacteremia (A) Kidneys were inspected for surface abscesses. A1: PBS treatment group. A2: Low concentration celastrol (6.25 mg/kg) treatment group. A3: Middle concentration celastrol (12.5 mg/kg) treatment group. A4: High concentration celastrol (25 mg/kg) treatment group. A5: Linezolid (25 mg/kg) treatment group. (B) Bacterial burdens in the livers of different groups. NS, not significant; *$p < 0.05$, compared with PBS treatment group.

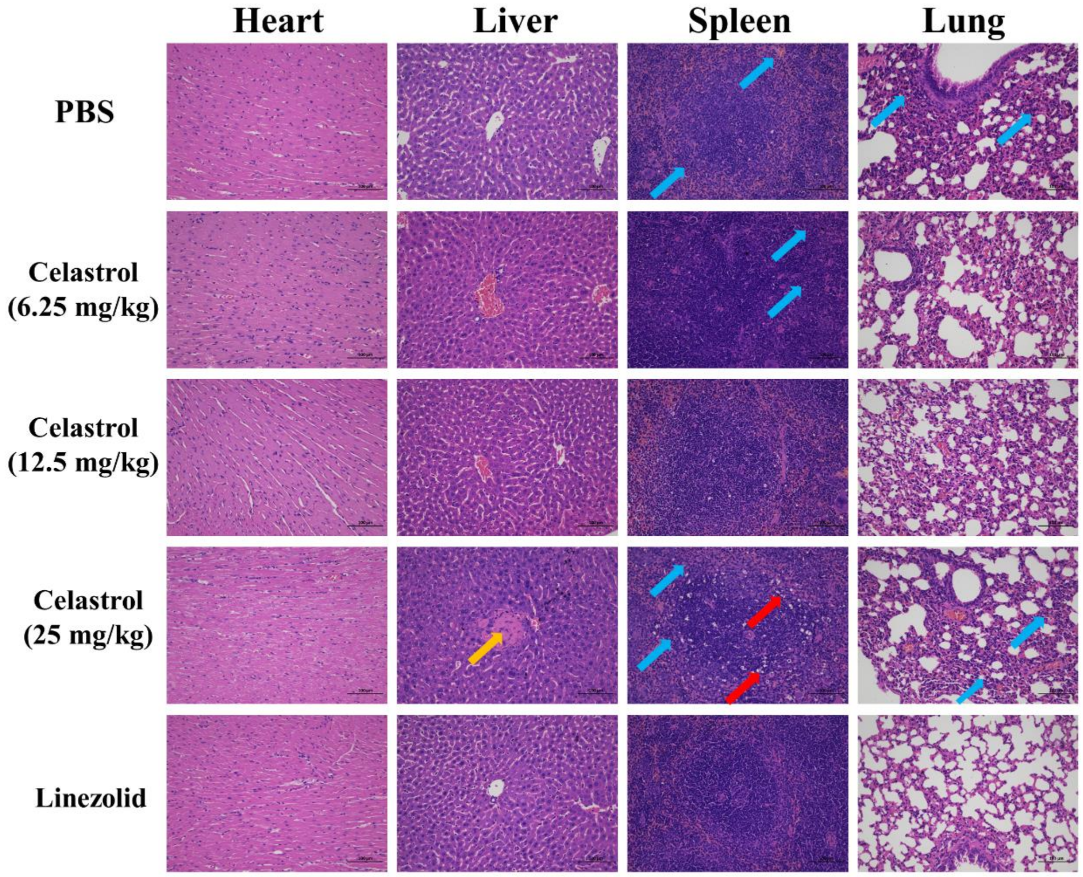

Figure S2. H&E staining on different organs (heart, liver, spleen, and lung) from the infected mice with different treatments. Blue arrows: inflammatory cell infiltration; Red arrows: cell apoptosis; Yellow arrows: cell necrosis.

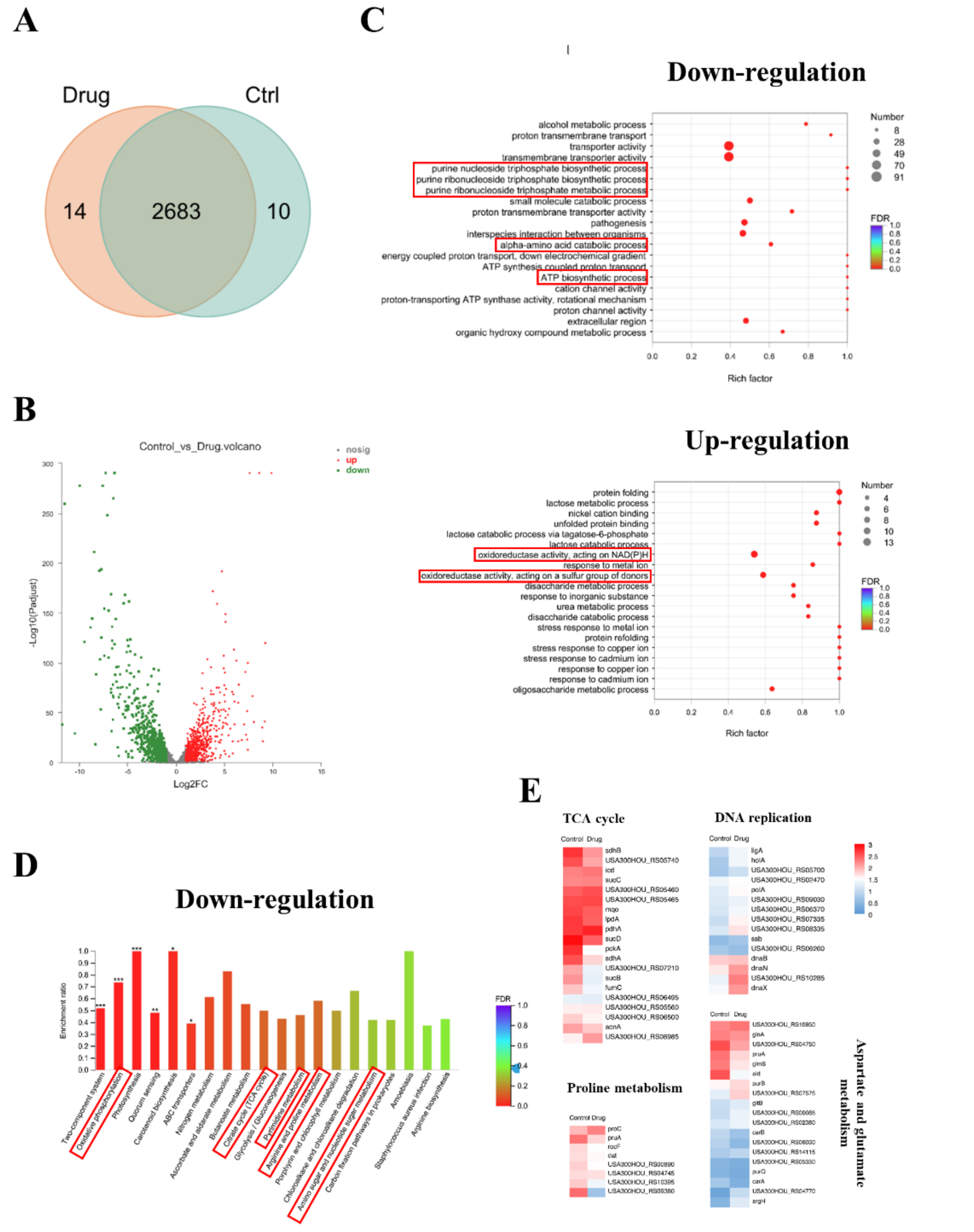

Figure S3. Transcriptomic analysis of MRSA USA300 treated with celastrol or DMSO. (A) Venn diagram. (B) The distribution of differentially expressed genes (DEGs) are presented in volcano plot. (C) GO enrichment analysis. (D) KEGG enrichment analysis. The x and y axis in a represent the expression changes and corresponding statistically significant degree, respectively. (E) Selected DEGs involved in TCA cycle, DNA replication, proline metabolism, and glutamate and aspartate metabolism. Drug: celastrol; Control: DMSO.

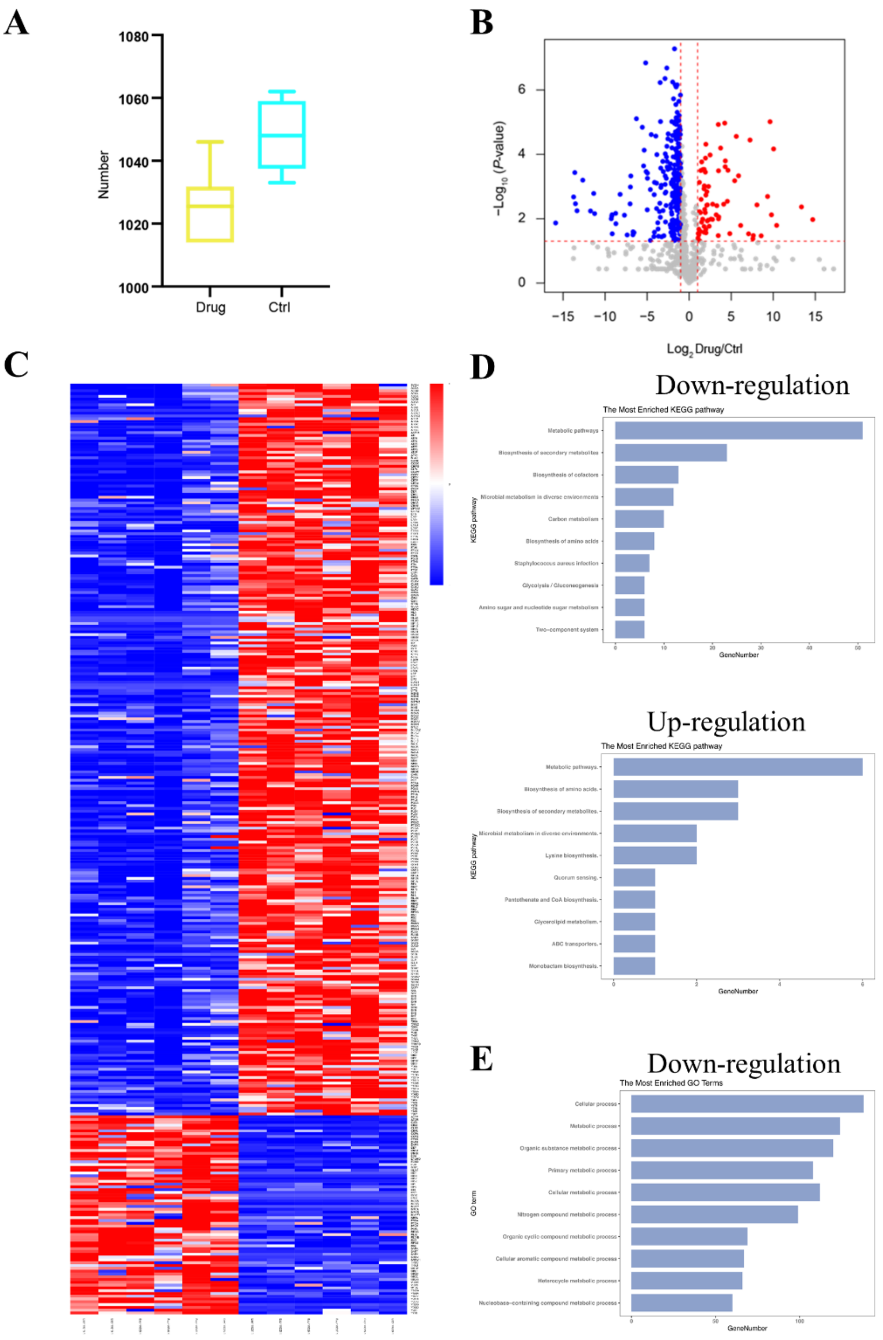

Figure S4. Proteomics analysis of MRSA USA300 treated with celastrol or DMSO. (A) Box plot of protein identification. (B) The distribution of differentially expressed proteins are presented in volcano plot. (C) Cluster heat map of some important DEPs. (D) KEGG enrichment analysis. (E) GO enrichment analysis. Drug: celastrol; Ctrl: DMSO.

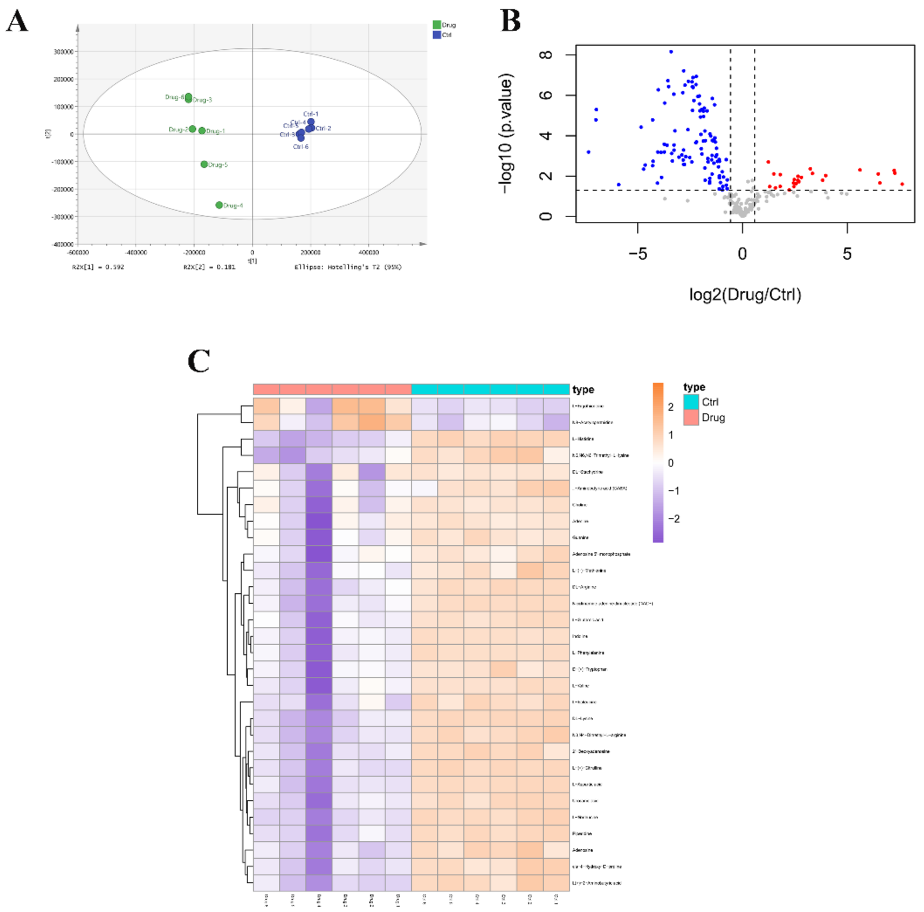

Figure S5. Metabolomics analysis of MRSA USA300 treated with celastrol or DMSO (A) Principal Component Analysis. (B) Volcano plot. (C) Overview metabolic profile heat map. Drug: celastrol; Ctrl: DMSO.

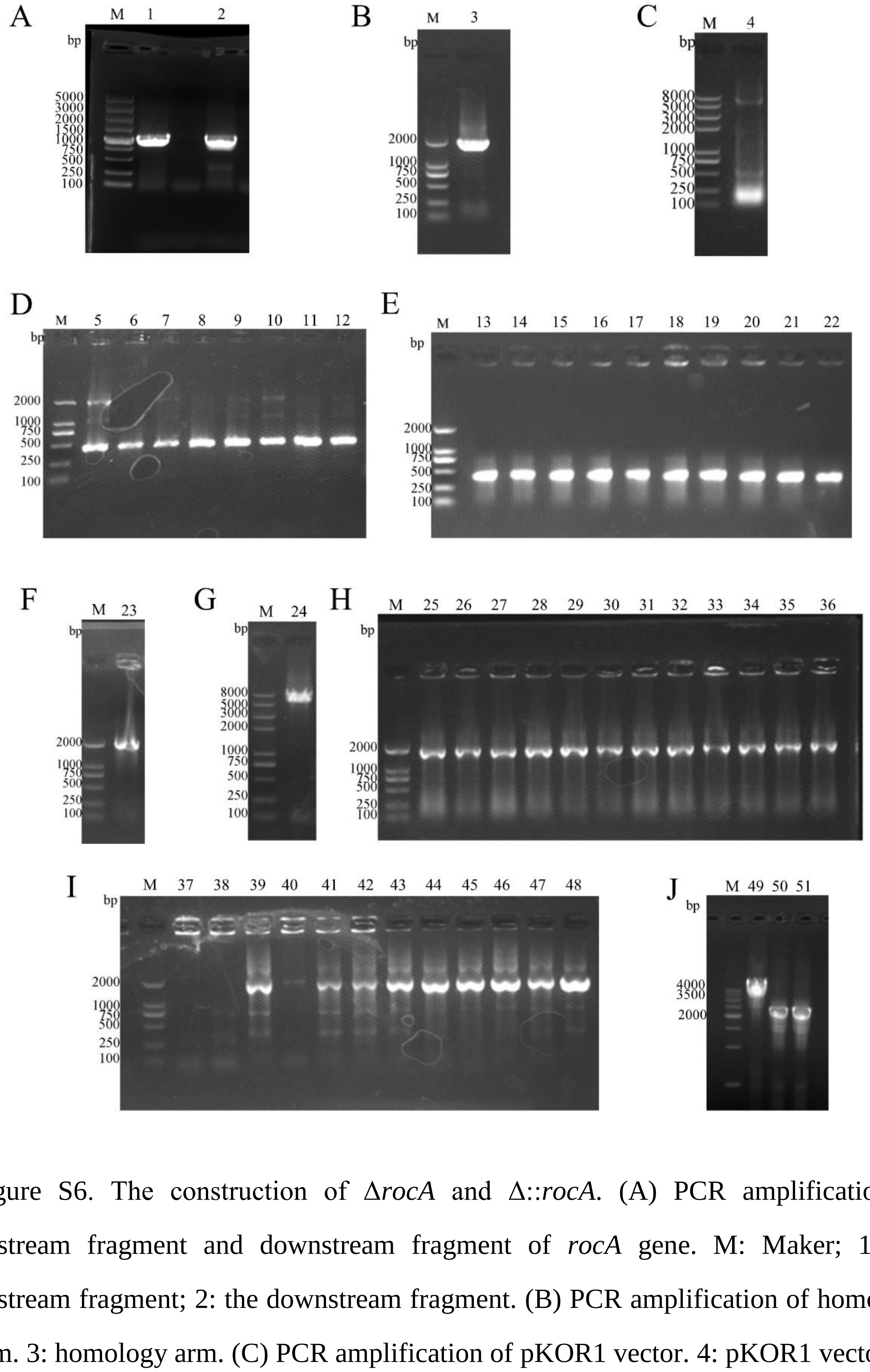

Figure S6. The construction of Δ*rocA* and Δ::*rocA*. (A) PCR amplification of upstream fragment and downstream fragment of *rocA* gene. M: Maker; 1: the upstream fragment; 2: the downstream fragment. (B) PCR amplification of homology arm. 3: homology arm. (C) PCR amplification of pKOR1 vector. 4: pKOR1 vector. (D) PCR identification of RN4220 Δ*rocA*. 5-12: randomly selected colony clones. (E) PCR identification of MRSA USA300 Δ*rocA*. 13-22: randomly selected colony clones. (F) PCR amplification of *rocA* gene with promoter. 23: *rocA* gene with

promoter. (G) PCR amplification of pCM28 vector. 24: pCM28 vector. (H): PCR identification of pCM28-rocA vector in DH5α. 25-36: randomly selected colony clones. (I): PCR identification of transformed pCM28-rocA vector in RN4220 strain. 37-48: randomly selected colony clones. (J): PCR identification of the Δ*rocA* and Δ::*rocA*. 49: WT; 50: Δ*rocA*; 51: Δ::*rocA*.

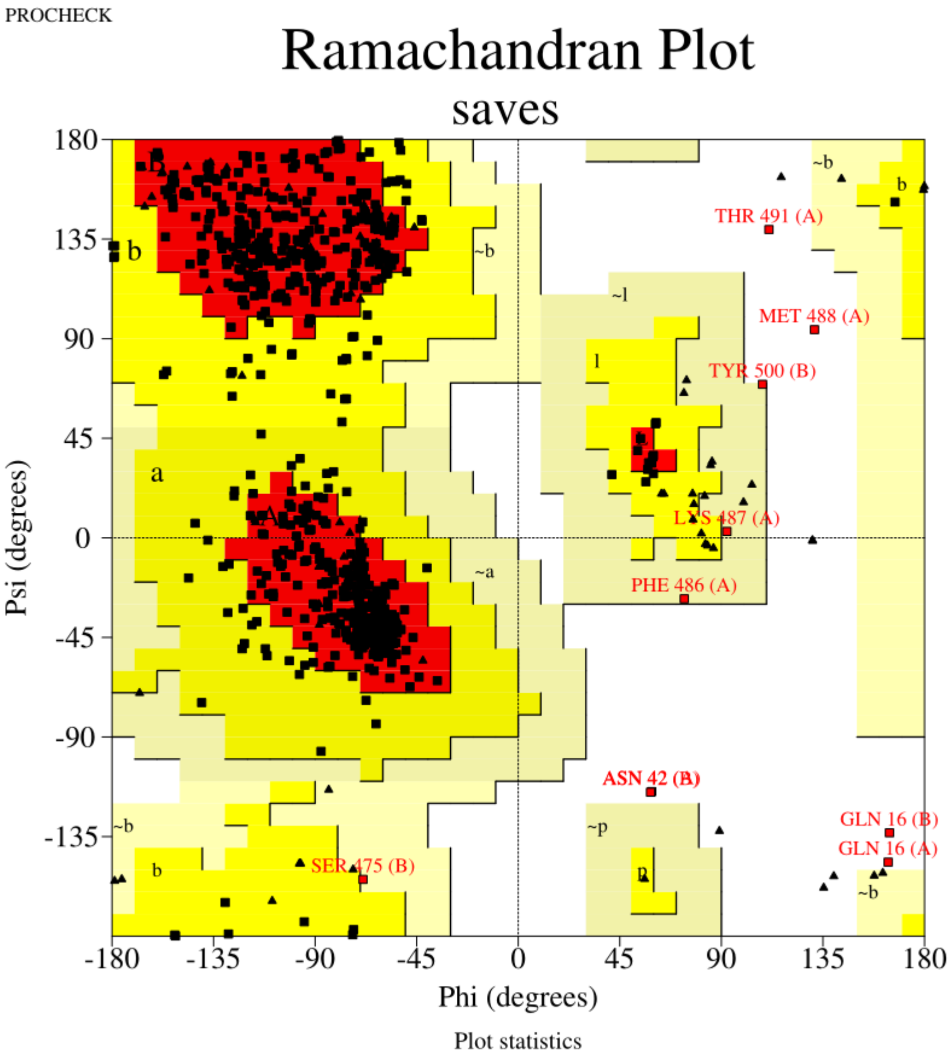

Figure S7. Quality assessment of P5CDH proteins

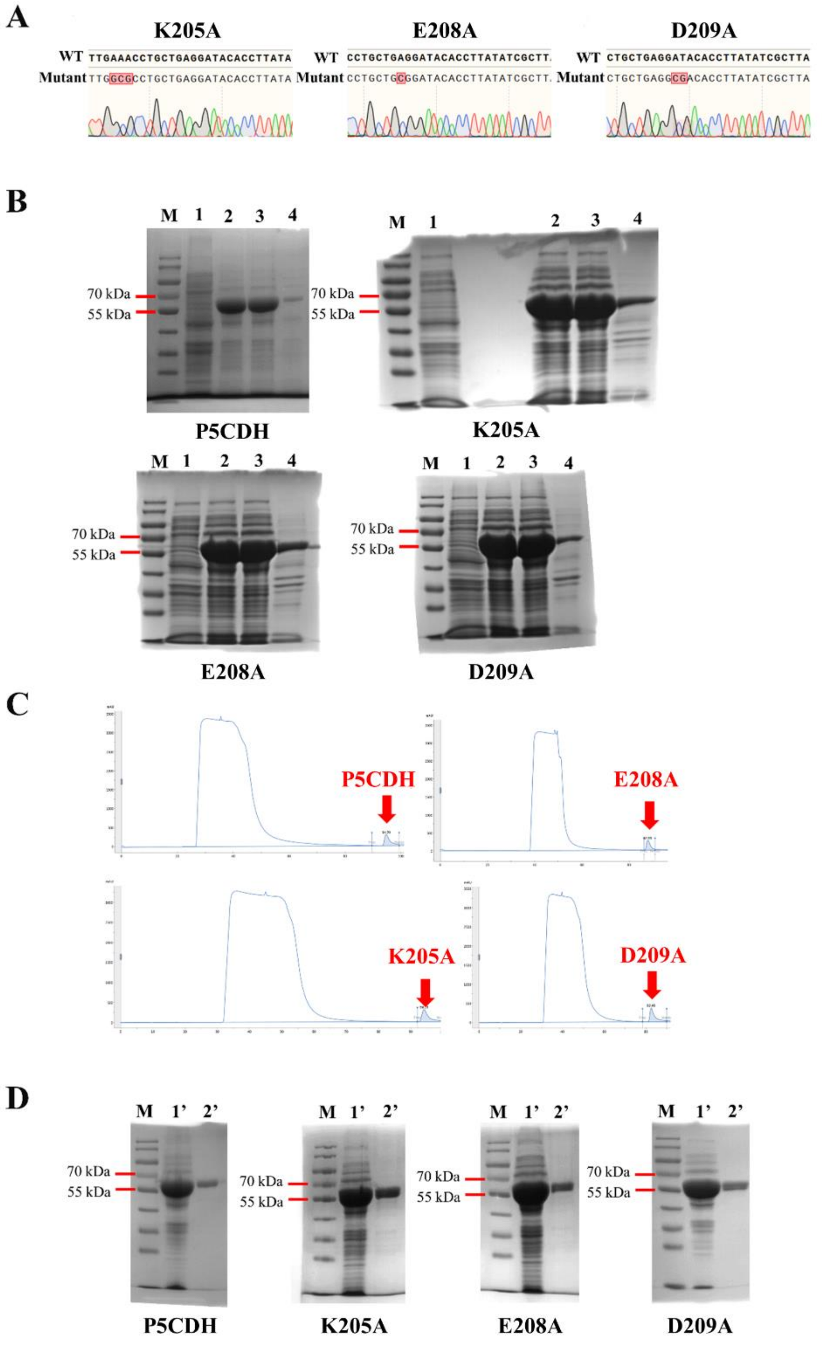

Figure S8. The expression and purification of four proteins (P5CDH, K205A, E208A and D209A) (A) A representative sequencing chromatogram of three P5CDH mutant proteins. (B) SDS-PAGE gel analysis of P5CDH and its mutant proteins expression as induced by the presence or absence IPTG. M: Protein molecular mass marker; lanes 1: The lysates of BL21 (DE3) cells without IPTG; lanes 2: The lysates of BL21 (DE3) cells with IPTG; lanes 3: The supernatants of BL21 (DE3) cells with IPTG; lanes 4:

The precipitates of BL21 (DE3) cells with IPTG. (C) SDS-PAGE gel analysis of P5CDH and its mutant proteins expression with or without purification. M: Protein molecular mass marker; lanes 1': The supernatants of BL21 (DE3) cells without purification; lanes 2': The supernatants of BL21 (DE3) cells after purification. (D) AKTA purified protein absorption curve of P5CDH and its mutant proteins.

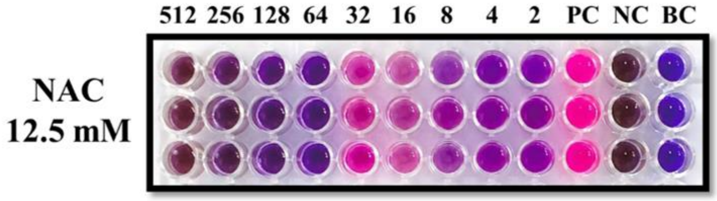

Figure S9 Synergistic activity of celastrol in combination with NAC (N-acetylcysteine) against MRSA USA300

Supplementary Tables

Table S1 Strains and plasmids used in this study

Strains or plasmids	Description	Source/Reference
**Strains**		
Staphylococcus aureus ATCC BAA-1717 (USA300)	Standard strain	ATCC
Staphylococcus aureus ATCC 43300	Standard strain	ATCC
Staphylococcus aureus RN4220	Standard strain (Restriction-deficient transformation recipient)	ATCC
Staphylococcus aureus 5ZB12	Clinical strain	Lab stock
Staphylococcus aureus 2ZG3	Clinical strain	Lab stock
Staphylococcus aureus 5ZB14	Clinical strain	Lab stock
Staphylococcus aureus 25FS35	Clinical strain	Lab stock
Staphylococcus aureus HX86	Clinical strain	Lab stock
Staphylococcus aureus YFC28	Clinical strain	Lab stock
Staphylococcus aureus HB119	Clinical strain	Lab stock
Staphylococcus aureus 25FS24	Clinical strain	Lab stock
Staphylococcus aureus 26FS31	Clinical strain	Lab stock
Staphylococcus aureus 6Y2C	Clinical strain	Lab stock
Staphylococcus aureus 7SX2	Clinical strain	Lab stock
USA300 Δ*rocA*	*rocA* knockout strain	This study
USA300 Δ:: *rocA*	*rocA* complemented strain	This study
Escherichia coli DH5α	Used to amplify plasmids	Lab stock
Escherichia coli BL21(DE3)	Used to expression of target proteins	Lab stock
Staphylococcus aureus ATCC 25923	Standard strain	ATCC
Staphylococcus aureus ATCC 29213	Standard strain	ATCC
Staphylococcus xylosus ATCC 29971	Standard strain	ATCC
Staphylococcus xylosus ATCC 700404	Standard strain	ATCC
Streptococcus suis ATCC 700794	Standard strain	ATCC
Streptococcus suis D2	Clinical strain	Lab stock
Streptococcus suis D29	Clinical strain	Lab stock
Streptococcus suis F2-4	Clinical strain	Lab stock
Streptococcus suis F8-3	Clinical strain	Lab stock
Escherichia coli ATCC 25922	Standard strain	ATCC

Escherichia coli FS24	Clinical strain	Lab stock
Acinetobacter baumannii B136	Clinical strain	Lab stock
Acinetobacter baumannii 3F2-AB3	Clinical strain	Lab stock
Klebsiella pneumonia E-4C2	Clinical strain	Lab stock
Klebsiella pneumonia E-4H3	Clinical strain	Lab stock
**Plasmids**		
pET30a	For Protein expression	Commercially available
pET30a-P5CDH	pET30a derivative for P5CDH expression	This study
pET30a-P5CDH- K205A	pET30a derivative for P5CDH ΔLys205 expression	This study
pET30a-P5CDH- E208A	pET30a derivative for P5CDH ΔGlu208 expression	This study
pET30a-P5CDH- D209A	pET30a derivative for P5CDH ΔAsp209 expression	This study
pKOR1	*S.aureus* genome editing vector	Hangzhou forhighbio
pKOR1-rocA	pKOR1 with upstream and downstream of the *rocA* gene	This study
pCM28	For gene completation	Hangzhou forhighbio
pCM28-rocA	pCM28 with *rocA* sequence	This study

ATCC, American Type Culture Collection.

Table S2. Primers used in this study

Name	Sequence (5'-3')	Description
Protein expression		
P5CDH-F	AAGGAGATATACATATGATGGTAGTAGAATTTAAAAATGAACCTGGTTACGA	P5CDH protein expression
P5CDH-R	GGTGGTGGTGCTCGAGGAACATTTCTGAAACAACCTTTTGTTCTAAGAAGTG	
Gene deletion		
WFKL-F	ATACCCATGGTCTAGATACTCACATTGTACGTTAATAGATTAAAAAATGAATGCGT	Amplification of totally 1kb rocA upstream
UP-R	GTTCATTATGAAGCAGTACACTTACCCCCTATAAATTTTGAAAGTGGT	
WFKL-R	ATTTTTCTGACTCGAGGTAGATTATTGTTCGATTATAGTATCTATCCCCGACC	amplification of totally 1kb rocA downstream
DOWN-F	TAGGGGGTAAGTGTACTGCTTCATAATGAACAAGGGCCATCC	
pKOR1-rocA-F	AATACTACAGACGCATTCAATTGCGTTGCGCTCACTGCCC	Amplification of totally 2kb rocA upstream and downstream for gene deletion
pKOR1-rocA-R	AATCGAACAATAATCTACGGTACCGGTTCCGAGGCTCAAC	
rocA-pKOR1-F	CCTCGGAACCGGTACCGTAGATTATTGTTCGATTATAGTA	Amplification of the vector
rocA-pKOR1-R	GAGCGCAACGCAATTGAATGCGTCTGTAGTATTGAATTT	
rocA-JD-F	CCGCTATCTAAATGACTTTGACT	To verify the knock strain
rocA-JD-R	ATTGATAATGACCTACCATCCGA	
WJD-F	TAGCAAGTGGTATCAATATTGCAGCTACAA	To verify the knock strain from upstream and downstream
WJD-R	ATTATTAAGATGGATGACTTTATCATCATCTTTGTTGTCT	
rocA-F	ATGGTAGTAGAATTTAAAAATGAACCTGGTTACGAT	To verify the knock strain
rocA-R	TTAGAACATTTCTGAAACAACCTTTTGTTCTAAGAAG	
Gene complementation		

pCM-rocA-F	GTTTCAGAAATGTTCTAAGAATTCGTAATCATGTCATAGC	Amplification of rocA sequence for gene completation
pCM-rocA-R	TATATGGGTCGCAACTGAAGCTTTTAAAAAGCAAATATGA	
rocA-pCM-F	ATTTGCTTTTTAAAAGCTTCAGTTGCGACCCATATAAATT	Amplification of the vector
rocA-pCM-R	ATGACATGATTACGAATTCTTAGAACATTTCTGAAACAAC	
